# Temperature-controlled optical response characteristic of an ionic liquid-based neodymium complex

**DOI:** 10.3389/fchem.2026.1888956

**Published:** 2026-08-21

**Authors:** Shiqi Zhao, Boxiao Zhang, Shuai Liu, Wenjie Song, Xinnuo Zhao, Yibo Wang, Chunyan Zhou

**Affiliations:** 1 Department of Chemistry, Beijing Technology and Business University, Beijing, China; 2 Department of Physics, Beijing Technology and Business University, Beijing, China; 3 Department of Pharmacy, First Affiliated Hospital of Dalian Medical University, Dalian, China

**Keywords:** coordination polymer, lanthanide, luminescence, near-infrared, temperature sensing

## Abstract

Lanthanide-based metal–organic coordination polymers (Ln-MOCPs) have been widely used in luminescent temperature sensing. Among them, Nd^3+^ has become a highly promising luminescent center due to its intense radiative transition properties in the first and second near-infrared regions. In this work, {[NdIMDC(H_2_O)_4_]Cl_2_.2H_2_O}_*n*_ (NdIMDC, IMDC = C_7_H_7_N_2_O_4_) was synthesized using Brønsted acidic ionic liquid, 1,3-bis(carboxymethyl)imidazolium chloride ([H_2_IMDC]Cl), as light-harvester. The crystal structure of NdIMDC was characterized by single-crystal X-ray diffraction (SCXRD), its composition and morphology were determined by fourier transform infrared spectroscopy (FT-IR), powder X-ray diffraction (PXRD), and scanning electron microscopy (SEM), and its thermal stability is evaluated by thermogravimetric analysis (TGA). The temperature-dependent luminescence properties of NdIMDC were assessed with the relative sensitivity (*Sr*) and temperature uncertainty (Δ*T*) of 0.618% K^−1^ and ±0.29 K. These findings confirm that NdIMDC establishes a well-defined linear relationship between its characteristic emission intensity ratio and temperature, suggesting its promising application in near-infrared luminescent temperature sensing.

## 1 Introduction

With the increasing demand for fast, precise and non-invasive temperature detection in fields such as engineering materials, biological imaging, and extreme environment monitoring and so on, the development of high-sensitivity luminescent temperature sensing materials has received widespread attention (Ding et al., 2021). Compared with traditional temperature measurement methods, luminescent temperature sensing technology has shown significant advantages due to its non-contact measurement, rapid response, high spatial resolution, and excellent applicability in complex electromagnetic fields and dynamic environments (Wang et al., 2021). In various luminescent sensing systems, characteristic signals such as luminescence intensity, spectral position, and luminescence lifetime of materials can undergo regular changes with external temperature, thereby achieving quantitative characterization of temperature. Compared with sensing strategies that rely exclusively on a single signal readout, a rationally designed luminescent system delivers strong immunity to external interferences including light source intensity fluctuations, photobleaching, and analyte concentration discrepancies, thus enhancing both the reliability and signal-to-noise ratio of temperature measurement outcomes (Zhao et al., 2015). To date, extensive research efforts in the field of luminescent temperature sensing have been dedicated to a diverse range of material platforms, spanning upconversion nanoparticles, metal chelates, core-shell nanostructured materials, organic fluorescent molecules, quantum dots, perovskites, and metal-organic coordination polymers (MOCPs) (Zhao et al., 2020). MOCPs are crystalline hybrid materials constructed by self-assembly, in which metal nodes (metal ions or metal clusters) are closely linked with organic linkers through coordination bonds (Hu et al., 2021; Liu et al., 2023). These materials feature highly ordered structures and good thermal stability, exhibiting great application potentials in sensing, catalysis, optoelectronic materials and so on (Patra and Pal, 2025). The coordination environment, electronic transition, and energy relaxation processes of metal centers in MOCPs are highly responsive to external temperature variations. This inherent characteristic endows them with distinct advantages for high-sensitivity, non-invasive luminescent temperature sensing applications, including intracellular temperature monitoring and fault diagnosis of microelectronic devices (Da Silva et al., 2025).

Owing to the unique 4*f*–4*f* electronic transition properties, lanthanide ions (Ln^3+^) exhibit sharp characteristic emission peaks, large Stokes/anti-Stokes shifts, long luminescence lifetimes, excellent photostability, and luminescence covering the visible to the second near-infrared region (Fan et al., 2025; Luo et al., 2017). It is well established that the luminescence of Ln^3+^ is generally sensitized through “antenna effect” of organic light-harvesting ligands, and then the luminescence performance respond correspondingly to the variation of ambient temperature (Chen et al., 2016; Wang et al., 2019). Such response sensitivity is usually reflected in measurable variations in luminescent intensity, emission color or lifetime, thereby enabling highly accurate temperature measurement. In addition, the transition energy within the 4*f* energy level is nearly unaffected by the surrounding chemical environment (including ligands, host crystals, and solvents), which enables reliable assignment of excitation and emission spectra as well as accurate prediction of inter-level energy differences (De souza et al., 2022). Thus, Lanthanide-based materials show remarkable advantages in biological imaging, optical sensing materials (Lian et al., 2015; Zhou et al., 2018). Among them, NIR luminescent MOCPs have several outstanding characteristics. First, in comparison with ultraviolet excitation sources, near-infrared excitation sources deliver a remarkably greater tissue penetration depth, induce negligible damage to living organisms, and enhance both the signal-to-noise ratio and sensitivity in biological detection scenarios (Zhang et al., 2023). Second, Nd^3+^ exhibits characteristic near-infrared luminescence properties, with its emission bands falling exactly within the first and second biological windows (Li et al., 2022). Moreover, Nd^3+^ has abundant electronic energy levels with moderate energy intervals, and the radiative transitions and non-radiative relaxation processes of different transition energy levels display significant sensitivity to temperature variation. With varying external temperature, the intensities of different characteristic emission peaks of Nd^3+^ show distinct changing trends, providing a reliable optical signal basis for the construction of temperature-sensing models with high stability and signal-to-noise ratio (Gomez et al., 2020; Suta et al., 2020). However, Nd-based crystals with different skeleton structures and ligand types exhibit differentiated thermal sensing performance due to differences in the local coordination field strength, lattice vibration, and micro polarity environment around Nd^3+^. In reported studies, Da Silva et al. (2025) developed a nanoscale Nd-MOCP with a ligand of terephthalic acid as a sensor in the second biological window, and its *Sr* at 298 K reach to 0.13% K^−1^ (Da Silva et al., 2025). Carlos et al. proposed using Nd^3+^-doped lanthanum oxychloride nanocrystals as an efficient system, with *Sr* (for 2% Nd^3+^) of 0.26% K^−1^ (Carrasco et al., 2015). In the Nd-MOCP system, organic ligands not only connect Nd^3+^ to form a highly ordered structure, but also achieve efficient incident light absorption and energy transfer (ET) through the “antenna effect”, significantly enhancing the near-infrared luminescence efficiency of Nd^3+^. Furthermore, the conjugated structure, coordination mode, and structural rigidity of organic ligands directly also affect the energy transfer efficiency, non-radiative relaxation rate, and luminescent thermal response behaviors of the Ln-MOCPs system (Luo et al., 2017). Traditional multidentate carboxylic acids are excellent ligands for synthesizing coordination polymers, which can form structures with various topologies and conformations. Carboxylic acid groups can act as monodentate, bidentate, or multidentate ligands to coordinate with metal centers (Wang et al., 2020). Although Nd-MOCPs constructed based on traditional organic ligands can achieve near-infrared temperature sensing function, the maximum relative sensitivity of most materials is not particularly high, and these systems are generally accompanied by considerable temperature measurement uncertainty (Carrasco et al., 2015; Da Silva et al., 2025; Kolesnikov et al., 2017). The above phenomenon fully demonstrates that relying solely on traditional organic ligands to construct or physically mix Nd-based crystals is difficult to achieve high-sensitivity and high-precision near-infrared output synchronously. Therefore, the rational design of ligand structures by introducing different groups plays a crucial role in improving the luminescent performance and temperature response sensitivity of Nd-MOCP.

Among the wide variety of organic light-harvesting ligands, ionic liquids (ILs) — characterized by their low volatility, high thermal stability, excellent structural designability, and strong interionic interactions—are considered as ideal functional units for regulating the structure, light-harvesting properties, and energy transfer efficiency of metal-organic materials (Yamada and Mizuno, 2021; Yang et al., 2025; Yang et al., 2026; Yin et al., 2022). Consequently, the design and synthesis of suitable *π*-conjugated ILs as “antenna” ligands represents a judicious strategy for constructing Ln-MOCPs. Conventional ILs are typically employed solely as solvents or modifying phases and do not participate in metal coordination. Functional coordination ILs can directly participate in the construction of metal center coordination environments, compensating for the shortcomings of traditional carboxylic acid ligands in micro coordination environment and optical property. Research indicates that ILs based on imidazolium cations are the most extensively utilized, and numerous studies have grafted various functional groups onto these cations to tailor ILs with distinct physicochemical properties. According to our previous research (Yang et al., 2025; Yang et al., 2026; Yang et al., 2023; Yin et al., 2022), Brønsted acidic ILs, due to its symmetrical planar conjugated structure, exhibit high light-harvesting and suitable triplet level, thereby endowing Ln-MOCPs with outstanding luminescent characteristics (Yin et al., 2022). The designed and synthesized Brønsted acidic ILs, when incorporated into Ln-MOCPs, generate abundant charges within the structural framework and thus form strong electrostatic interactions, which effectively enhance the stability of the resulting Ln-MOCPs. The synergistic effect between Ln^3+^ and Brønsted acidic IL ligands can effectively suppress the aggregation of IL chromophores, mitigate luminescence quenching, and reduce non-radiative energy dissipation. Our previous studies have corroborated the aforementioned points: Brønsted acidic ILs based on imidazolium cations demonstrate numerous advantages in constructing luminescent Ln-MOCPs materials, including highly structural symmetry and rigidity, good thermal stability, readily coordinating carboxylic acid groups, high light-harvesting capability and ET efficiency, all of which significantly improve the crystallinity, stability, and mechanical strength of Ln-MOCPs materials (AL-Aqmar et al., 2017; Wang et al., 2019). These advantages of Brønsted acidic ILs are more conducive to fabricating well-defined ordered structures, which enhance the energy transfer efficiency and quantum yield of these materials, optimize the near-infrared luminescent performance of Nd^3+^, and thereby offer a new approach for constructing high-performance near-infrared temperature sensing materials.

In this study, utilizing carboxyl-functionalized Brønsted acidic ILs as light-harvesting linker, we have constructed a novel structurally stable luminescent Ln-MOCPs material, {[NdIMDC(H_2_O)_4_]Cl_2_.2H_2_O}_*n*_ (NdIMDC, IMDC = C_7_H_7_N_2_O_4_), via a solvothermal method. We have systematically characterized the crystal structure and composition, phase purity, thermal stability, and near-infrared luminescent properties. Furthermore, an investigation into its luminescent response behavior within a specified temperature range was conducted. By analyzing the intensity variations of the characteristic emission peaks of NdIMDC, we successfully demonstrated a highly sensitive temperature-responsive luminescent performance.

## 2 Experimental section

### 2.1 Experimental materials and testing methods

With the exception of [H_2_imdc]Cl, all reagents and chemical samples used in the experiment were commercially purchased and do not require further purification.

The ^1^H nuclear magnetic resonance (^1^H NMR) spectrum and ^13^C nuclear magnetic resonance (^13^C NMR) spectrum were acquired on a Bruker AM 400 MH NMR analyzer using dimethyl sulfoxide (DMSO) as the solvent and tetramethylsilane (TMS) as the standard. Fourier transform infrared (FTIR) spectra were recorded using a Nicolet Avatar 360 FTIR spectrometer in transmission mode from 500 to 4,000 cm^−1^. Thermogravimetric analysis (TGA) of the substances was carried out on a Mettler-Toledo TGA/DSC 3+ analyzer with a dry-air background and a temperature rise rate of 10 °C/min from 35 °C to 700 °C. Scanning electron microscopy (SEM) images of the NdIMDC(H_2_O)_4_ surface were obtained by JEM-6700E, JEOL. Powder X-ray diffraction data were collected using a Bruker D8 ADVANCE with Cu Kα radiation (*λ* = 1.5406 Å). Measurements were made in a 2*θ* range of 8–50° at room temperature with a step of 0.05° (2*θ*). The operating power was 40 kV and 30 mA. Inductively coupled plasma spectroscopy (ICP) was recorded on an ICPE-9000 spectrometer. The emission spectra at room temperature were recorded with a Hitachi F-7000 fluorescence spectrometer. The temperature-dependent emission spectra of the complexes were measured on a NanoLog TCSPC spectrometer from Horiba Jobin Yvon. Single-crystal X-ray diffraction data for the coordination polymers were collected on a Bruker APEX-II CCD diffractometer equipped with graphite monochromatic Ga Kα radiation (*λ* = 1.34139 Å). Using Olex2, the structure was solved with the SHELXT structure solution program using Intrinsic Phasing and refined with the SHELXL refinement package using Least Squares minimization.

### 2.2 Synthesis and ^1^H NMR of [H_2_IMDC]Cl

[H_2_IMDC]Cl was synthesized with reference to the literature in the yield of 87.4% (Fei et al., 2004). ^1^H NMR: 9.27 (s, 1H, C(2)H), 7.79 (s, 2H, C(4,5)H), 5.26 ppm (s, 4H, NCH_2_). ^13^C NMR: 168.49 (s, 2C, COOH), 138.75 (s, 1C, C(2)H), 123.89 (s, 2C, C(4, 5)H), and 50.34 ppm (s, 2C, NCH_2_).

### 2.3 Synthesis of {[NdIMDC(H_2_O)_4_]Cl_2_·2H_2_O}_*n*_

A mixture of NdCl_3_.6H_2_O (0.1 mmol), [H_2_IMDC]Cl (0.1 mmol), NaOH (0.3 mol/L, 0.40 mL), 5.0 mL isopropanol and 5.0 mL chloroform were placed into a 23.0 mL stainless steel autoclave with a PTFE (Teflon) liner at room temperature. The autoclave was sealed and heated at 140 °C for 48 h. After the reaction was completed, light purple blocky single crystals {[NdIMDC(H_2_O)_4_]Cl_2_·2H_2_O}_*n*_ were obtained by cooling to room temperature and filtration. Building upon this foundation, we systematically optimized the molar ratio of NdCl_3_·6H_2_O to [H_2_IMDC]Cl and fine-tuned the solvent conditions through iterative adjustments. Run 1–4 was carried out at a fixed metal-to-ligand molar ratio of 2:1 with only the amount of NaOH and solvent ratio adjusted, showing generally better crystallization quality than other groups. Among them, Run 4 yielded regular, transparent purple bulk single crystals, exhibiting the best crystallization performance. In contrast, the other three runs primarily produced polycrystals, flaky clusters, and fragmented crystals, which were markedly inferior in both integrity and quality. Runs 5 and 6 were conducted with a metal-to-ligand molar ratio of 1:1. However, varying the NaOH and solvent ratios yielded primarily microcrystals, powders, and fragmented grains. Consequently, intact single crystals could not be obtained, resulting in a marked decline in crystallization quality. In Runs 7–9, the reactant molar ratio was further adjusted to 1:1.5. Excess IMDC ligand sharply accelerated the nucleation rate, yielding polycrystalline powders, fragmented crystals, and irregular aggregates. A comprehensive comparison demonstrates that only the parameter combination employed in Run 4 facilitates stable and ordered crystal growth. Deviations from these optimal conditions cause uncontrolled crystallization, yielding products with markedly inferior morphology and quality. The results indicate that the optimized reaction conditions resulted in significantly improved morphology and purity, as detailed in Supplementary Table S1.

Elemental analysis for C_7_H_9_N_2_O_4_Cl (220.45): calculated C 38.104, H 4.083, N 12.701; found C 37.580, H 4.273, N 12.960. Elemental analysis for C_7_H_19_N_2_NdO_10_Cl_2_ (506.38): calculated C 16.588, H 3.752, N 5.529; found C 16.474, H 3.862, N 5.432. The elemental analysis of organic ligands H_2_IMDC]Cl and target coordination polymers NdIMDC shows a high degree of agreement between the measured C, H, and N element contents and the theoretically calculated values, proving the successful preparation of NdIMDC.

## 3 Results and discussion

### 3.1 Characterizations of {[NdIMDC(H_2_O)_4_]Cl_2_·2H_2_O}_*n*_

**FIGURE 1:**
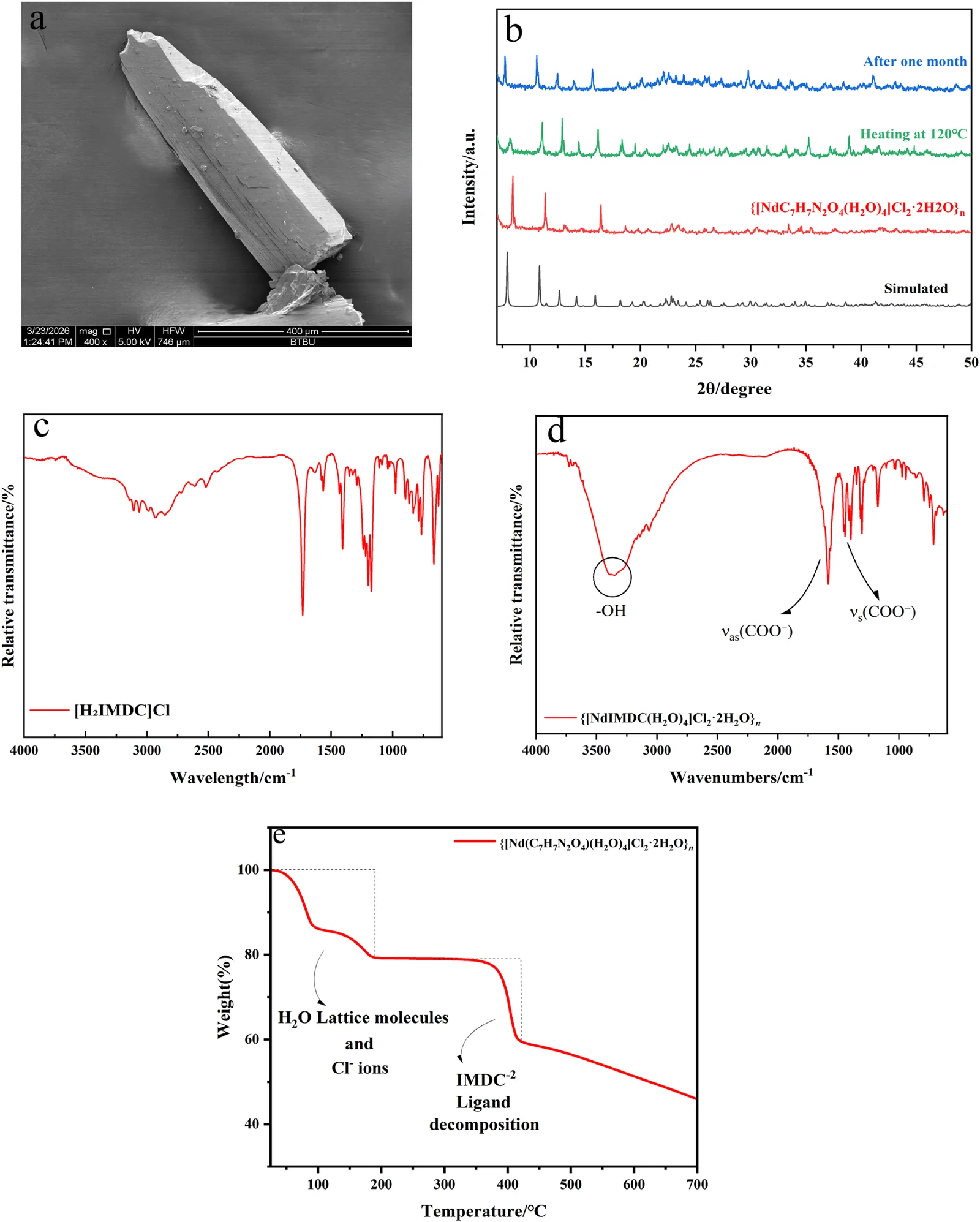
**(a)** SEM images of NdIMDC, **(b)** Powder X-ray diffraction pattern and simulated diffraction pattern of NdIMDC, **(c)** FT-IR spectra of [H_2_IMDC]Cl, **(d)** FT-IR spectra of NdIMDC, **(e)** Thermogravimetric analysis curves for {[Nd(C_7_H_7_N_2_O_4_)(H_2_O)_4_]Cl_2_·2H_2_O}_*n*_.

The SEM image of {[NdIMDC(H_2_O)_4_]Cl_2_.2H_2_O}_*n*_ is shown in Figure 1a, with a long columnar shape of the as-synthesized NdIMDC crystals with the overall length of about 580 μm. Parallel and continuous layered growth steps formed by layer by layer stacked molecular units can be observed on the crystal side, with no large-area pores, cracks, or amorphous impurities, proving that the ligand and Nd^3+^ coordination assembly process of this system is highly regular. This verifies the highly regular ligand-Nd^3+^ coordination assembly, excellent crystal growth orientation and high crystal purity of the products. A powder X-ray diffraction (PXRD) pattern exhibits sharp diffraction peaks, as shown in Figure 1b. The positions and intensities of the diffraction peaks in the experimental PXRD pattern are consistent with the simulated pattern derived from single-crystal data, indicating that the sample has high crystallinity and good phase purity.

The FT-IR spectrum of [H_2_IMDC]Cl is highly consistent with that reported in the literature (Liu et al., 2022) (Figure 1c). The presence of the imidazolium moiety [H_2_IMDC]Cl is easily confirmed by vibrations ranging from 3,200 to 2,800 cm^−1^, assigned to methylene groups (ν_asym_[–CH_2_]) attached to the imidazolium ring and C–H of imidazolium ring. The strong absorption peak at approximately 1730 cm^-1^ is the characteristic peak of the *ν*_as_(-COOH) stretching vibration of carboxylic acid, confirming the presence of carboxylic acid groups in [H_2_IMDC]Cl. The absorption peak at 1,562 cm^-1^ is assigned to the skeletal vibration of the imidazole ring. The absorption peak at 1,406 cm^-1^ is attributed to *ν*_s_(-COOH) the stretching vibration of carboxylic acid groups. The peak at 1,176 cm^-1^ belongs to the stretching vibration of the imidazole ring. The absorption peaks in the range of 500–1,000 cm^-1^ are mainly attributed to the in-plane bending vibrations of C–H bonds. The above FT-IR results are highly consistent with the expected structure, proving that [H_2_IMDC]Cl has been successfully synthesized.

The broad and strong absorption peak in the range of 3,650–3,000 cm^-1^ in Figure 1d is attributed to the O–H stretching vibration of coordinated water and O–H‧‧‧Cl hydrogen bonds in NdIMDC. The absence of the strong absorption peak at 1730 cm^-1^ indicates that the carboxyl groups are completely deprotonated and no free carboxyl groups exist. The characteristic absorption peak around 1,586 cm^-1^ is not only attributed to the asymmetric stretching vibration of the carboxyl group, but also overlaps with the C–N stretching vibration of the imidazole ring skeleton. The absorption peak around 1,399 cm^-1^ is attributed to the symmetric stretching vibration of the carboxyl group, and the appearance of these two groups of peaks proves that the carboxyl groups of the ligand have been completely deprotonated. The absorption peak appearing at 1,450–1,470 cm^-1^ is the deformation vibration of C–H in the imidazole ring. Several medium-intensity peaks in the range of 1,304–1,038 cm^-1^ are mainly caused by various bending vibrations in the C–H plane. The absorption peaks in the far-infrared region of 600–500 cm^-1^ correspond to the stretching vibration of Nd–O, indicating the successful coordination between [IMDC]^-^ and Nd^3+^, thereby realizing the synthesis of the target material (Da Silva et al., 2025).

For the thermogravimetric analysis (TGA) of NdIMDC (Figure 1e), the first mass loss of 20.65% (calculated: 21.13%) occurs at 55 °C–185 °C, which is attributed to the weight loss of lattice water and two chloride ions. As the temperature rises to 350 °C, the framework begins to collapse, resulting in the second mass loss. When the temperature rises to 700 °C, the complex has been completely converted into the lanthanide oxide Nd_2_O_3_.

### 3.2 Crystal structural description

**FIGURE 2:**
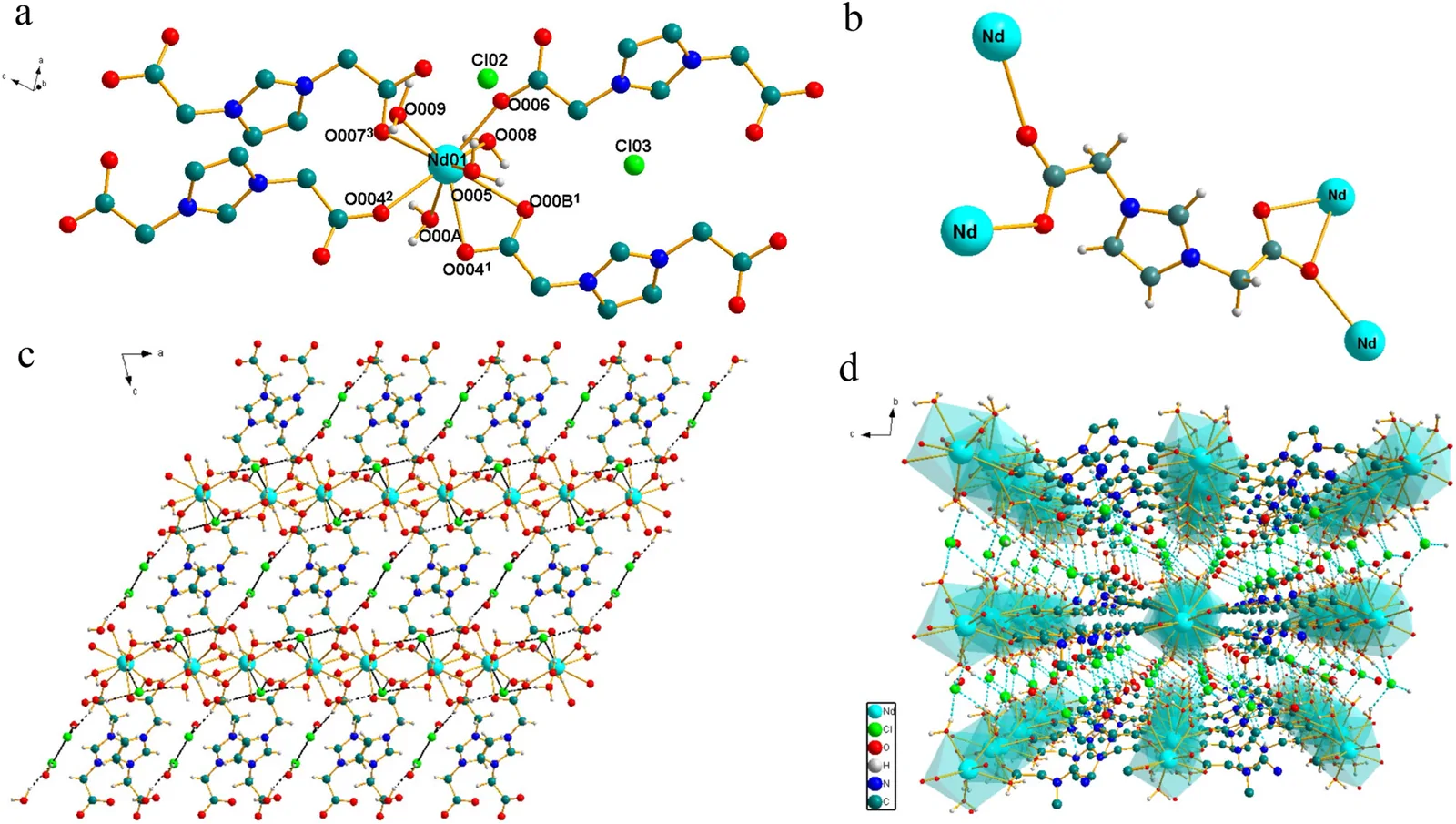
**(a)** Coordination environment of Nd^3+^, **(b)** Coordination mode of [IMDC]^−^, **(c)** 2D layer parallel to *ac* plane, **(d)** 3D framework of NdIMDC viewed along the *b* axis. For clear views, some H atoms are omitted and H-bonds are shown as dotted lines.

The crystal structure data of {[NdIMDC(H_2_O)_4_]Cl_2_·2H_2_O}_*n*_ (CCDC:2551106) are listed in Supplementary Table S2. This table lists the key crystallographic parameters extracted from the standard CIF (Crystallographic Information File), including atomic coordinates and crystal packing information. Some bond lengths, bond angles, and hydrogen bond data of {[NdIMDC(H_2_O)_4_]Cl_2_·2H_2_O}_*n*_ is shown in Supplementary Tables S3–S5. In NdIMDC, Nd^3+^ ions display only one crystallographically independent chemical environment, as illustrated in Figure 2a. Nd^3+^ is surrounded by four oxygen atoms (O005, O008, O009, and O00A) from coordination water and five carboxyl oxygen atoms (O004^1^, O004^2^, O006, O007, and O00B) from four IMDC, forming a nine-coordination chemical environment. The bond lengths of Nd−O range from 2.416 to 2.642 Å, with an average bond length of approximately 2.52 Å, which is consistent with the distance found in analogous neodymium carboxylate complexes (Luo et al., 2017; Pan et al., 2016). In NdIMDC, [IMDC]^-^ adopts only one coordination mode as shown in Figure 2b. Two carboxyl groups are both deprotonated: one carboxyl group binds to two Nd^3+^ through a bridging bidentate mode; the other connects two Nd^3+^ via a chelating-bridging tridentate mode. Two carboxyl groups from two [IMDC]^-^ link two adjacent Nd^3+^ together through coordinate bonds, with the shortest Nd···Nd distance of 4.3912 Å, thus constructing the Nd_2_(COO)_4_ secondary building unit. Subsequently, adjacent secondary building units form one-dimensional chains (1D) along *a* axis, and then two-dimensional (2D) layers are formed by 1D chains through Nd−O_carboxyl_, which is parallel to *ac* plane (Figure 2c). Cl^−^ ions from the reactants form intensive hydrogen bonds with the coordination and lattice H_2_O. Eventually, a three-dimensional architecture is fabricated by 2D layers through hydrogen bonds (Cl···H-O) and electrostatic interactions between positively charged framework and lattice Cl^−^ (Figure 2d). This significantly enhances the structural stability of NdIMDC, which can be observed in TGA curve in Figure 1e. Consistent with the earlier observation, in IL-based Ln-MOCPs, the charge compensation of the positively charged framework can usually be achieved by ions such as Cl^−^ or Br^−^ present in the reactants or solvents (Yang et al., 2025).

### 3.3 Temperature-dependent luminescent properties of NdIMDC

**FIGURE 3:**
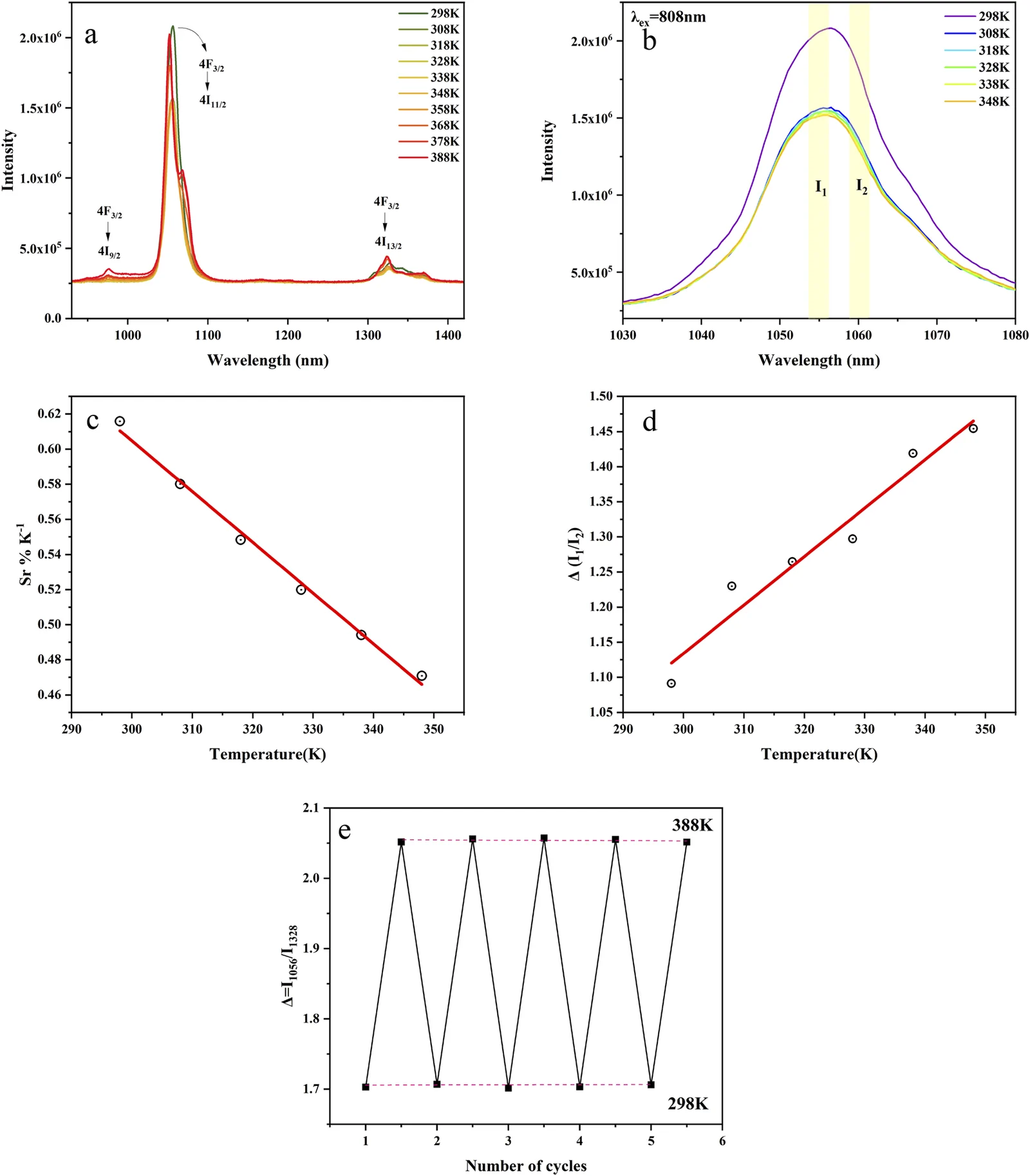
**(a)** Emission spectrum in temperature range of 298–388 K, **(b)** Emission spectrum in temperature range of 298–348 K, **(c)** Temperature dependence intensity ratio of *Δ* = *I*_1_ */I*_2_, **(d)** Relative thermal sensitivity *Sr* for NdIMDC (*λ*_ex_ = 808 nm), **(e)** The reversible emission intensity ratio (*I*_1056_/*I*_1328_) of the NdIMDC between 298 K and 388 K.

The luminescent performance of NdIMDC under 808 nm excitation was analyzed. The emission spectrum shows three characteristic emission peaks, corresponding to three energy level transitions of Nd^3+^ (^4^F_3_/_2_→^4^I_9_/_2_, ^4^F_3_/_2_→^4^I_11_/_2_, and ^4^F_3_/_2_→^4^I_13_/_2_) located at 977 nm, 1,056 nm, and 1,328 nm, respectively (Figure 3a). Among them, the emission peak at 1,056 nm is the highest and lies in the NIR-II bio-optical window, which is of great significance due to its excellent tissue penetration ability. Meanwhile, the TGA results (Figure 1e) showed that the skeletal structure of NdIMDC remained stable in the temperature range below 350 °C, and the as-synthesized NdIMDC exhibited excellent thermal stability. After heating to 120 °C or remaining under quiescent conditions for more than 1 month, the PXRD results showed (Figure 1b) that no significant variation was observed in both the emission peak position and intensity for these two NdIMDC samples. It indicates that the crystal structure of NdIMDC stayed nearly unaltered, while its phase purity was well preserved, which endows the material with great potential for a temperature sensing application.

In the variable-temperature near-infrared luminescence test from 298 to 388 K, the intensities of characteristic emission peaks exhibited different response patterns to temperature changes. Specifically, the intensity of the emission peak at 977 nm exhibited a continuous, monotonically increasing trend as the temperature rose gradually. While the two groups of emission peaks at 1,056 nm and 1,328 nm exhibited more complex temperature response behaviors. In the range of 298–358 K, the emission intensities at 1,056 nm and 1,328 nm gradually decreased with the increase of temperature. However, when the temperature was further increased from 358 K to 388 K, the emission peak intensity shifted to an upward trend, revealing an overall non-monotonic pattern characterized by an initial decrease followed by an increase (Figure 3b). The observed temperature-dependent luminescence behavior involves three core processes: ligand-to-Nd^3+^ ET in NdIMDC, an intrinsic Stark level distribution of Nd^3+^, and temperature-responsive non-radiative relaxation (Marciniak et al., 2024). These processes are strongly influenced by the system’s phonon energy and the tested temperature range (Puccini et al., 2024; Rocha et al., 2016). In addition, the intensity change of a single transition in the fine structure is usually caused by the rearrangement of the number of electrons in the Stark sub-levels of the excited state of the Ln^3+^ (Gomez et al., 2020). So, in order to effectively distinguish the various transition components and quantitatively analyze their intensity variation patterns, this work performs peak fitting on the luminescence spectra.

Upon 808 nm excitation, [IMDC]^-^ absorbs excitation energy as an acceptor, and then energy efficiently transfers the T_1_ state energy to the Nd^3+^, thereby sensitizing Nd^3+^ to produce characteristic near-infrared emission. For the ^4^F_3_/_2_→^4^I_9_/_2_ transition, the corresponding terminal state energy level structure has higher stability, lower sensitivity to lattice thermal vibration, and less influence by external temperature. At present, it is known that the ET of [IMDC]^-^ to Nd^3+^ exhibits dynamic characteristics. In the temperature range of 298–388K, the phonon-assisted [IMDC]^-^→Nd^3+^ ET process dominates. The elevation of temperature corresponds to an increase in phonon density, which effectively bridges the energy gap between [IMDC]^-^ and Nd^3+^ (Chen et al., 2020; Jaque et al., 2003). On the one hand, through lattice thermal expansion and coordination bond relaxation effects, the excited state energy levels of [IMDC]^-^ are induced to undergo thermal displacement; On the other hand, intensified lattice vibrations induce subtle fluctuations in the coordination environment, leading to a degree of thermal broadening of these excited-state levels of [IMDC]^-^. These two effects work together to increase the effective energy overlap between the excited state of [IMDC]^-^ and the Nd^3+^ acceptor level, optimize the energy level matching, and significantly improve the forward energy transfer efficiency from [IMDC]^-^ to Nd^3+^ (Abbas et al., 2026). Throughout this process, positive energy transfer dominates, effectively improving the electron distribution on the metastable 4F_3/2_ level of Nd^3+^, significantly increasing the probability of radiative transitions, and ultimately resulting in a sustained increase in emission peak intensity with increasing temperature (Jiang et al., 2022).

For two transitions of ^4^F_3_/_2_→^4^I_11_/_2_ and ^4^F_3_/_2_→^4^I_13_/_2_, the corresponding energy level are more susceptible to the thermal vibration of NdIMDC framework and changes in the coordination microenvironment, and there are obvious systematic differences in the temperature response mechanism. Within the temperature range of 298 K–358 K, the increase of temperature will gradually intensify the lattice vibration of LnIMDC framework. On the one hand, it promotes the energy dissipation of excited state through non-radiative relaxation; on the other hand, it induces the reverse ET from Nd^3+^ to the [IMDC]^-^ to a certain degree, weakening the effective luminescence intensity (Gomez et al., 2020). Under the dual effects, the intensities of two transitions in this temperature range continue to decrease. When the temperature gradually increased to 388 K, the originally clear and sharp spectral lines showed thermal broadening in the spectrum, accompanied by the appearance of shoulder peaks and a shift in spectral position. This situation may be attributed to the thermal activation effect, which led to the removal of lattice water, blocking multi-phonon relaxation channels, and thus allowing more excited Nd^3+^ to release energy through radiative transitions (Chen et al., 2020). Consequently, the thermal activation effect is significantly enhanced, and forward energy transfer once again becomes the dominant process (Yue et al., 2016). With increasing temperature, the phonon-assisted ET process from [IMDC]^-^ to Nd^3+^ gradually becomes dominant, and its probability increases accordingly (Abbas et al., 2026). The phenomenon is also confirmed by the TGA curve, which presents the first weigh loss at about 85 °C. This not only improves the energy transfer efficiency from [IMDC]^-^ to the Nd^3+^ center, but also promotes the rearrangement of electrons on different Stark sub-levels of Nd^3+^, effectively offsetting the luminescence quenching caused by non-radiative relaxation. Finally, the intensities of the emission peaks at 1,056 nm and 1,328 nm gradually rise.

Although the intensities of three groups emission peaks show different mechanisms with temperature changes, the luminescence intensity ratio of the characteristic emission peaks corresponding to different Stark sub-levels of Nd^3+^ still shows a good linear relationship. This phenomenon indicates that although different transition channels are affected by thermal perturbations through distinct pathways, their relative intensity changes still follow a predictable pattern, reflecting the inherent stability of energy level coupling within the system and providing a reliable basis for ratio-based optical temperature sensing.

^4^F_3_/_2_→^4^I_11_/_2_ transition of Nd^3+^ does not produce a single spectral line; instead, it consists of multiple sub-peaks derived from transitions between different Stark sublevels. The emission spectra of different Stokes energy levels were recorded at temperatures ranging from 298 to 358 K (Supplementary Figure S1). Among them, the Stoke energy levels at 1,054 nm and 1060 nm were denoted as *I*_1_ and *I*_2_, respectively. These two sub peaks originate from the same energy level, and the source of thermal response is consistent, without signal interference caused by different transitions. And their corresponding spectral regions have clear boundaries and no significant overlap. In order to accurately analyze these components, Gaussian functions were used to analyze the emission spectra, as shown in the equation:

$$y=y_{0}+\frac{A}{W\sqrt{\pi /\left(4\ln\left(2\right)\right)}}\exp\left(-\frac{4\ln\left(2\right){\left(x-x_{c}\right)}^{2}}{w^{2}}\right)$$

By selecting the intensity ratio of two sub-peaks as the temperature-sensing parameter, we utilized the different energy losses induced by lattice thermal vibrations at different sub-energy levels, which result in varying rates of luminescence intensity decay and differences in temperature sensitivity, to establish a quantitative temperature measurement calibration curve. This difference exhibits predictable and temperature-dependent variations that effectively mitigate external interferences, including instrument fluctuations and excitation power instability, thereby providing a robust quantitative foundation for ratiometric optical temperature sensing. The intensity ratio of two transition at 1,054 nm and 1,060 nm, defined as *Δ* = *I*_1_/*I*_2_, and the difference in attenuation speed between the two sub-peaks enables *Δ* to exhibit a stable monotonic variation with temperature (R^2^ = 0.942) in the temperature range of 298–348 K, and *Sr* gradually decreases with increasing temperature, reaching a maximum value of 0.618% K^−1^ at 298 K (Figures 3c,d). Their relationship can be expressed by the following formula:

$$I_{1}/I_{2}=‒0.93583+0.0069T$$

In general, neodymium-based optical thermometers without sensitizers (such as Yb^3+^) to boost Nd^3+^ emission signals typically exhibit *Sr​* values below 1%. Nevertheless, NdIMDC achieves *Sr​* of 0.618% K^−1^ and *ΔT* of ±0.29 K, which are superior to other unsensitized neodymium-based materials and comparable to neodymium-based MOCPs employing sensitizers, while boasting a simple and rapid synthesis process (Da Silva et al., 2025) (Supplementary Table S6). To verify the stability and reversibility of NdIMDC as thermal sensing material, temperature cycling data were obtained after the material was stored for half a month (Figure 3e). After five heating-cooling cycles, the luminescence intensity ratio remained stable, indicating the reusability of NdIMDC in temperature sensing.

## 4 Conclusion

The carboxyl-functionalized N-trimethylsilylimidazole ionic liquid was employed to synthesize 1,3-bis(carboxymethyl)imidazolium chloride (H_2_[IMDC]Cl). Using H_2_[IMDC]Cl as a dual-functional organic ligand for light-harvesting and sensitization, a 3D supramolecular LnIMDC was successfully prepared through the solvothermal method. In NdIMDC, the distinct temperature responses of the three characteristic Nd^3+^ NIR emission peaks allow for a maximum relative sensitivity (*S*_r_) of 0.618% K^−1^ at 298 K and a temperature resolution (*ΔT*) of ±0.29 K in the 298–348 K range. Despite the drawbacks of solvothermal single-crystal synthesis (high-temperature/high-pressure conditions and relatively low product yield), and fluorescence intensity of the Nd^3+^characteristic emission peak changes relatively gently during the heating process, their near-infrared emission peaks fall exactly within the second biological window. The mild thermal decay behavior can prevent signal quenching in the biological microenvironment, making it suitable for long-term non-destructive *in-situ* biological temperature measurement. In addition, the light-harvesting and sensitization effects of [IMDC]^−^ endow the material with excellent cycling stability, without thermal cycling-induced phase separation issues, enabling it to be used for real-time tracking of the operating temperature of various thin films and electrolyte devices (Zhang et al., 2023). This work successfully introduces structurally tailorable ILs into photoluminescent functional materials, and expands the applications of ILs and their functional coordination complexes in the field of optical temperature sensing.

## Data Availability

The raw data supporting the conclusions of this article will be made available by the authors, without undue reservation.
