# Supplementary material for: Temperature-controlled optical response characteristic of an ionic liquid-based neodymium complex
Source: Front Chem. 2026 Aug 21;14:1888956. doi: 10.3389/fchem.2026.1888956 (PMC13541709; doi:10.3389/fchem.2026.1888956)
Supplement: Supplementary file 1 [file DataSheet1.pdf]

Temperature-Controlled Optical Response Characteristic of an Ionic Liquid-Based Neodymium Complex

Shiqi Zhao[a], Boxiao Zhang[a], Wenjie Song[a], Xinnuo Zhao[a], Chunyan Zhou[b*], Yibo Wang[a*]

[a]*Department of Chemistry, Beijing Technology and Business University, Beijing 100048, China*

[b]*First Affiliated Hospital of Dalian Medical University, Dalian, 116011, China*

Corresponding Authors

[*]*E-mail address: wangyb@th.btbu.edu.cn (Y. Wang)*

$\{[NdC_7H_7N_2O_4(H_2O)_4]Cl_2{\cdot}2H_2O\}_n$

data_nd

_audit_creation_date 2026-05-01

_audit_creation_method

;

Olex2 1.5

(compiled 2024.02.16 svn.r378c4104 for OlexSys, GUI svn.r6928)

;

_shelx_SHELXL_version_number '2018/3'

_audit_contact_author_address ?

_audit_contact_author_email ?

_audit_contact_author_name ''

_audit_contact_author_phone ?

_publ_contact_author_id_orcid ?

_publ_section_references

;

Dolomanov, O.V., Bourhis, L.J., Gildea, R.J, Howard, J.A.K. & Puschmann, H.
(2009), J. Appl. Cryst. 42, 339-341.

Sheldrick, G.M. (2015). Acta Cryst. A71, 3-8.

Sheldrick, G.M. (2015). Acta Cryst. C71, 3-8.

;

_chemical_name_common ?

_chemical_name_systematic ?

```
_chemical_formula_moiety                  'C7 H15 N2 Nd O8, 2(Cl), 2(H2 O)'
_chemical_formula_sum                      'C7 H19 Cl2 N2 Nd O10'
_chemical_formula_weight                  506.38
_chemical_melting_point                   ?
loop_
    _atom_type_symbol
    _atom_type_description
    _atom_type_scat_dispersion_real
    _atom_type_scat_dispersion_imag
    _atom_type_scat_source
  'C' 'C' 0.0137 0.0067 'International Tables Vol C Tables 4.2.6.8 and 6.1.1.4'
  'Cl' 'Cl' 0.3281 0.5435 'International Tables Vol C Tables 4.2.6.8 and 6.1.1.4'
  'N' 'N' 0.0241 0.0134 'International Tables Vol C Tables 4.2.6.8 and 6.1.1.4'
  'Nd' 'Nd' -1.2627 8.7680
  'International Tables Vol C Tables 4.2.6.8 and 6.1.1.4'
  'O' 'O' 0.0389 0.0241 'International Tables Vol C Tables 4.2.6.8 and 6.1.1.4'
  'H' 'H' 0.0000 0.0000 'International Tables Vol C Tables 4.2.6.8 and 6.1.1.4'

_shelx_space_group_comment
;
The symmetry employed for this shelxl refinement is uniquely defined
by the following loop, which should always be used as a source of
symmetry information in preference to the above space-group names.
They are only intended as comments.
;
_space_group_crystal_system              'triclinic'
_space_group_IT_number                     2
_space_group_name_H-M_alt                 'P -1'
_space_group_name_Hall                    '-P 1'
loop_
    _space_group_symop_operation_xyz
  'x, y, z'
  '-x, -y, -z'
```

_cell_length_a 8.9977(5)
_cell_length_b 9.2732(5)
_cell_length_c 11.5128(6)
_cell_angle_alpha 89.849(2)
_cell_angle_beta 76.887(2)
_cell_angle_gamma 62.216(2)
_cell_volume 821.74(8)
_cell_formula_units_Z 2
_cell_measurement_reflns_used 8351
_cell_measurement_temperature 295.00
_cell_measurement_theta_max 57.07
_cell_measurement_theta_min 4.97
_shelx_estimated_absorpt_T_max 0.251
_shelx_estimated_absorpt_T_min 0.208
_exptl_absorpt_coefficient_mu 19.094
_exptl_absorpt_correction_T_max 0.7512
_exptl_absorpt_correction_T_min 0.4253
_exptl_absorpt_correction_type none
_exptl_absorpt_process_details
;
SADABS-2016/2 (Bruker,2016/2) was used for absorption correction.
wR2(int) was 0.1342 before and 0.0859 after correction.
The Ratio of minimum to maximum transmission is 0.5662.
The \l/2 correction factor is Not present.
;
_exptl_absorpt_special_details ?
_exptl_crystal_colour 'clear light colourless'
_exptl_crystal_colour_lustre clear
_exptl_crystal_colour_modifier light
_exptl_crystal_colour_primary colourless
_exptl_crystal_density_diffrn 2.047
_exptl_crystal_density_meas ?
_exptl_crystal_density_method ?
_exptl_crystal_description block

```
_exptl_crystal_F_000                 498
_exptl_crystal_size_max              0.12
_exptl_crystal_size_mid              0.1
_exptl_crystal_size_min              0.1
_exptl_transmission_factor_max       ?
_exptl_transmission_factor_min       ?
_diffrn_reflns_av_R_equivalents      0.0775
_diffrn_reflns_av_unetI/netI         0.0609
_diffrn_reflns_Laue_measured_fraction_full    0.991
_diffrn_reflns_Laue_measured_fraction_max    0.989
_diffrn_reflns_limit_h_max           11
_diffrn_reflns_limit_h_min           -11
_diffrn_reflns_limit_k_max           11
_diffrn_reflns_limit_k_min           -11
_diffrn_reflns_limit_l_max           14
_diffrn_reflns_limit_l_min           -14
_diffrn_reflns_number                13873
_diffrn_reflns_point_group_measured_fraction_full    0.991
_diffrn_reflns_point_group_measured_fraction_max    0.989
_diffrn_reflns_theta_full            53.594
_diffrn_reflns_theta_max             57.066
_diffrn_reflns_theta_min             4.973
_diffrn_ambient_temperature          295.00
_diffrn_detector_area_resol_mean     ?
_diffrn_measured_fraction_theta_full    0.991
_diffrn_measured_fraction_theta_max    0.989
_diffrn_measurement_device_type      'Bruker APEX-II CCD'
_diffrn_measurement_method           '\f and \w scans'
_diffrn_radiation_type               GaK\a
_diffrn_radiation_wavelength         1.34139
_diffrn_source                       ?
_diffrn_standards_number             0
_reflns_Friedel_coverage             0.000
_reflns_Friedel_fraction_full        .
```

_reflns_Friedel_fraction_max .
_reflns_number_gt 3197
_reflns_number_total 3348
_reflns_special_details
;
Reflections were merged by SHELXL according to the crystal
class for the calculation of statistics and refinement.

_reflns_Friedel_fraction is defined as the number of unique
Friedel pairs measured divided by the number that would be
possible theoretically, ignoring centric projections and
systematic absences.
;
_reflns_threshold_expression 'I > 2\s(I)'
_computing_cell_refinement 'SAINT V8.40B (?, 2016)'
_computing_data_collection ?
_computing_data_reduction 'SAINT V8.40B (?, 2016)'
_computing_molecular_graphics 'Olex2 1.5 (Dolomanov et al., 2009)'
_computing_publication_material 'Olex2 1.5 (Dolomanov et al., 2009)'
_computing_structure_refinement 'SHELXL 2018/3 (Sheldrick, 2015)'
_computing_structure_solution 'SHELXT 2018/2 (Sheldrick, 2018)'
_refine_diff_density_max 1.321
_refine_diff_density_min -1.430
_refine_diff_density_rms 0.192
_refine_ls_extinction_coef .
_refine_ls_extinction_method none
_refine_ls_goodness_of_fit_ref 1.085
_refine_ls_hydrogen_treatment constr
_refine_ls_matrix_type full
_refine_ls_number_parameters 209
_refine_ls_number_reflns 3348
_refine_ls_number_restraints 0
_refine_ls_R_factor_all 0.0381
_refine_ls_R_factor_gt 0.0365

_refine_ls_restrained_S_all 1.085

_refine_ls_shift/su_max 0.001

_refine_ls_shift/su_mean 0.000

_refine_ls_structure_factor_coef Fsqd

_refine_ls_weighting_details

'w=1/[\s^2^(Fo^2^)+(0.0453P)^2^+0.5890P] where P=(Fo^2^+2Fc^2^)/3'

_refine_ls_weighting_scheme calc

_refine_ls_wR_factor_gt 0.0864

_refine_ls_wR_factor_ref 0.0872

_refine_special_details ?

_olex2_refinement_description

;

1. Fixed Uiso

At 1.2 times of:

All C(H) groups, All C(H,H) groups

At 1.5 times of:

All O(H,H) groups

2.a Free rotating group:

O00D(H00P,H00Q), O00F(H00R,H00S)

2.b Rotating group:

O005(H00A,H00B), O008(H00C,H00D), O009(H00E,H00F), O00A(H00G,H00H)

2.c Secondary CH2 refined with riding coordinates:

C00J(H00J,H00K), C00L(H00M,H00N)

2.d Aromatic/amide H refined with riding coordinates:

C00I(H00I), C00K(H00L), C00M(H00O)

;

_atom_sites_solution_hydrogens mixed

_atom_sites_solution_primary dual

_atom_sites_solution_secondary ?

loop_

_atom_site_label

_atom_site_type_symbol

_atom_site_fract_x

_atom_site_fract_y

_atom_site_fract_z
_atom_site_U_iso_or_equiv
_atom_site_adp_type
_atom_site_occupancy
_atom_site_site_symmetry_order
_atom_site_calc_flag
_atom_site_refinement_flags_posn
_atom_site_refinement_flags_adp
_atom_site_refinement_flags_occupancy
_atom_site_disorder_assembly
_atom_site_disorder_group
Nd01 Nd 0.23012(2) 0.53046(2) 0.99037(2) 0.01415(10) Uani 1 1 d . . . . .
Cl02 Cl 0.72179(14) -0.01428(14) 0.84102(12) 0.0351(3) Uani 1 1 d . . . . .
Cl03 Cl 0.4096(3) 0.8104(2) 0.57827(18) 0.0687(5) Uani 1 1 d . . . . .
O004 O 1.0093(4) 0.4612(4) 0.1107(3) 0.0198(6) Uani 1 1 d . . . . .
O005 O 0.3349(4) 0.2541(4) 0.8711(3) 0.0276(7) Uani 1 1 d . . . . .
H00A H 0.338686 0.266485 0.797198 0.041 Uiso 1 1 d R U . . .
H00B H 0.440155 0.189975 0.871518 0.041 Uiso 1 1 d R U . . .
O006 O 0.5276(4) 0.4384(4) 0.8802(3) 0.0259(7) Uani 1 1 d . . . . .
O007 O 0.8051(4) 0.3588(4) 0.8035(3) 0.0244(7) Uani 1 1 d . . . . .
O008 O 0.2739(4) 0.7828(4) 0.9549(3) 0.0258(7) Uani 1 1 d . . . . .
H00C H 0.212868 0.839323 0.908532 0.039 Uiso 1 1 d R U . . .
H00D H 0.232486 0.846050 1.020494 0.039 Uiso 1 1 d R U . . .
O009 O 0.3806(4) 0.2949(4) 1.1007(3) 0.0264(7) Uani 1 1 d . . . . .
H00E H 0.355645 0.218553 1.087714 0.040 Uiso 1 1 d R U . . .
H00F H 0.502526 0.267142 1.087833 0.040 Uiso 1 1 d R U . . .
O00A O -0.0436(4) 0.7915(4) 1.0487(3) 0.0264(7) Uani 1 1 d . . . . .
H00G H -0.043904 0.843887 1.109769 0.040 Uiso 1 1 d R U . . .
H00H H -0.128685 0.772016 1.073530 0.040 Uiso 1 1 d R U . . .
O00B O 0.8127(4) 0.4054(4) 0.2206(3) 0.0251(7) Uani 1 1 d . . . . .
N00C N 0.9517(5) 0.3656(5) 0.4183(3) 0.0217(8) Uani 1 1 d . . . . .
O00D O 0.0947(5) 0.9586(4) 0.7960(4) 0.0356(8) Uani 1 1 d G . . . .
H00P H 0.165996 0.915610 0.727748 0.053 Uiso 1 1 d G U . . .
H00Q H 0.004656 0.953157 0.790737 0.053 Uiso 1 1 d G U . . .

N00E N 0.7859(5) 0.3381(5) 0.5740(3) 0.0258(8) Uani 1 1 d . . . . .

O00F O 0.3298(6) 0.1803(6) 0.6468(4) 0.0528(11) Uani 1 1 d G . . . .

H00R H 0.339927 0.085529 0.633699 0.079 Uiso 1 1 d G U . . .

H00S H 0.395613 0.195886 0.588473 0.079 Uiso 1 1 d G U . . .

C00G C 0.6595(5) 0.3896(5) 0.7936(4) 0.0183(8) Uani 1 1 d . . . . .

C00H C 0.9392(5) 0.4301(5) 0.2091(4) 0.0159(8) Uani 1 1 d . . . . .

C00I C 1.0511(7) 0.2189(6) 0.4562(4) 0.0320(12) Uani 1 1 d . . . . .

H00I H 1.167262 0.145430 0.421431 0.038 Uiso 1 1 calc R U . . .

C00J C 0.6328(6) 0.3712(7) 0.6699(4) 0.0317(11) Uani 1 1 d . . . . .

H00J H 0.605683 0.282114 0.664488 0.038 Uiso 1 1 calc R U . . .

H00K H 0.534525 0.471242 0.659206 0.038 Uiso 1 1 calc R U . . .

C00K C 0.7920(6) 0.4351(6) 0.4909(4) 0.0254(9) Uani 1 1 d . . . . .

H00L H 0.700027 0.535277 0.484187 0.030 Uiso 1 1 calc R U . . .

C00L C 1.0127(6) 0.4346(6) 0.3148(4) 0.0236(9) Uani 1 1 d . . . . .

H00M H 1.138463 0.373571 0.289113 0.028 Uiso 1 1 calc R U . . .

H00N H 0.980122 0.547512 0.339510 0.028 Uiso 1 1 calc R U . . .

C00M C 0.9469(7) 0.2022(6) 0.5539(5) 0.0389(13) Uani 1 1 d . . . . .

H00O H 0.978395 0.114505 0.599336 0.047 Uiso 1 1 calc R U . . .

loop_

_atom_site_aniso_label

_atom_site_aniso_U_11

_atom_site_aniso_U_22

_atom_site_aniso_U_33

_atom_site_aniso_U_23

_atom_site_aniso_U_13

_atom_site_aniso_U_12

Nd01 0.01331(14) 0.01615(15) 0.01255(15) 0.00345(9) -0.00094(9) -0.00775(10)

Cl02 0.0270(5) 0.0259(5) 0.0492(8) 0.0011(5) -0.0080(5) -0.0109(4)

Cl03 0.0746(11) 0.0599(10) 0.0612(11) -0.0165(8) 0.0207(9) -0.0405(9)

O004 0.0209(14) 0.0264(15) 0.0134(15) 0.0079(12) -0.0029(11) -0.0130(12)

O005 0.0219(15) 0.0255(16) 0.0300(18) -0.0007(13) -0.0050(13) -0.0076(13)

O006 0.0270(16) 0.0275(16) 0.0184(16) 0.0042(13) 0.0008(13) -0.0120(13)

O007 0.0235(15) 0.0323(17) 0.0220(16) 0.0020(13) -0.0070(12) -0.0165(13)

```
 O008 0.0246(15) 0.0238(15) 0.0294(18) 0.0085(13) -0.0076(13) -0.0118(13)
 O009 0.0206(14) 0.0275(16) 0.0359(19) 0.0089(14) -0.0080(13) -0.0151(13)
 O00A 0.0184(14) 0.0245(15) 0.0345(19) -0.0027(13) -0.0059(13) -0.0093(12)
 O00B 0.0260(15) 0.0382(17) 0.0213(16) 0.0106(13) -0.0075(12) -0.0230(14)
 N00C 0.0238(18) 0.032(2) 0.0136(17) 0.0060(15) -0.0057(14) -0.0166(16)
 O00D 0.0333(18) 0.0293(17) 0.043(2) -0.0028(16) -0.0071(16) -0.0156(16)
 N00E 0.0298(19) 0.038(2) 0.0135(18) 0.0056(16) -0.0043(15) -0.0194(17)
 O00F 0.061(3) 0.054(3) 0.042(3) -0.004(2) -0.008(2) -0.029(2)
 C00G 0.022(2) 0.0182(19) 0.016(2) 0.0040(16) -0.0020(16) -0.0116(16)
 C00H 0.0201(19) 0.0160(18) 0.0120(19) 0.0035(15) -0.0035(15) -0.0092(15)
 C00I 0.032(2) 0.031(2) 0.017(2) 0.0052(19) -0.0014(19) -0.004(2)
 C00J 0.036(3) 0.060(3) 0.013(2) 0.005(2) -0.0061(19) -0.034(3)
 C00K 0.024(2) 0.035(2) 0.017(2) 0.0069(18) -0.0072(17) -0.0130(18)
 C00L 0.028(2) 0.037(2) 0.012(2) 0.0071(18) -0.0035(17) -0.022(2)
 C00M 0.049(3) 0.030(2) 0.024(3) 0.009(2) 0.000(2) -0.011(2)

_geom_special_details
;
 All esds (except the esd in the dihedral angle between two l.s. planes)
 are estimated using the full covariance matrix.  The cell esds are taken
 into account individually in the estimation of esds in distances, angles
 and torsion angles; correlations between esds in cell parameters are only
 used when they are defined by crystal symmetry.  An approximate (isotropic)
 treatment of cell esds is used for estimating esds involving l.s. planes.
;
loop_
  _geom_bond_atom_site_label_1
  _geom_bond_atom_site_label_2
  _geom_bond_distance
  _geom_bond_site_symmetry_2
  _geom_bond_publ_flag
 Nd01 O004 2.642(3) 2_666 ?
 Nd01 O004 2.493(3) 1_456 ?
 Nd01 O005 2.554(3) . ?
```

Nd01 O006 2.416(3) . ?

Nd01 O007 2.481(3) 2_667 ?

Nd01 O008 2.564(3) . ?

Nd01 O009 2.495(3) . ?

Nd01 O00A 2.465(3) . ?

Nd01 O00B 2.570(3) 2_666 ?

Nd01 C00H 2.966(4) 2_666 ?

O004 C00H 1.268(5) . ?

O005 H00A 0.8530 . ?

O005 H00B 0.8530 . ?

O006 C00G 1.254(5) . ?

O007 C00G 1.237(5) . ?

O008 H00C 0.8508 . ?

O008 H00D 0.8515 . ?

O009 H00E 0.8587 . ?

O009 H00F 0.9776 . ?

O00A H00G 0.8542 . ?

O00A H00H 0.8543 . ?

O00B C00H 1.241(5) . ?

N00C C00I 1.371(6) . ?

N00C C00K 1.332(6) . ?

N00C C00L 1.468(5) . ?

O00D H00P 0.8500 . ?

O00D H00Q 0.8502 . ?

N00E C00J 1.459(6) . ?

N00E C00K 1.322(6) . ?

N00E C00M 1.374(7) . ?

O00F H00R 0.8500 . ?

O00F H00S 0.8500 . ?

C00G C00J 1.520(6) . ?

C00H C00L 1.519(6) . ?

C00I H00I 0.9300 . ?

C00I C00M 1.351(7) . ?

C00J H00J 0.9700 . ?

```
C00J H00K 0.9700 . ?
C00K H00L 0.9300 . ?
C00L H00M 0.9700 . ?
C00L H00N 0.9700 . ?
C00M H00O 0.9300 . ?

loop_
 _geom_angle_atom_site_label_1
 _geom_angle_atom_site_label_2
 _geom_angle_atom_site_label_3
 _geom_angle
 _geom_angle_site_symmetry_1
 _geom_angle_site_symmetry_3
 _geom_angle_publ_flag
O004 Nd01 O004 62.50(11) 1_456 2_666 ?
O004 Nd01 O005 82.15(10) 1_456 . ?
O004 Nd01 O008 138.91(10) 1_456 . ?
O004 Nd01 O009 71.89(10) 1_456 . ?
O004 Nd01 O00B 112.35(10) 1_456 2_666 ?
O004 Nd01 C00H 25.31(10) 2_666 2_666 ?
O004 Nd01 C00H 87.75(10) 1_456 2_666 ?
O005 Nd01 O004 69.12(10) . 2_666 ?
O005 Nd01 O008 136.13(10) . . ?
O005 Nd01 O00B 74.22(11) . 2_666 ?
O005 Nd01 C00H 68.63(11) . 2_666 ?
O006 Nd01 O004 123.12(10) . 2_666 ?
O006 Nd01 O004 148.32(9) . 1_456 ?
O006 Nd01 O005 73.16(11) . . ?
O006 Nd01 O007 107.51(11) . 2_667 ?
O006 Nd01 O008 71.47(10) . . ?
O006 Nd01 O009 80.46(10) . . ?
O006 Nd01 O00A 137.67(11) . . ?
O006 Nd01 O00B 80.02(10) . 2_666 ?
O006 Nd01 C00H 101.00(11) . 2_666 ?
```

O007 Nd01 O004 128.94(9) 2_667 2_666 ?

O007 Nd01 O004 78.51(10) 2_667 1_456 ?

O007 Nd01 O005 138.88(11) 2_667 . ?

O007 Nd01 O008 77.24(11) 2_667 . ?

O007 Nd01 O009 71.73(11) 2_667 . ?

O007 Nd01 O00B 146.87(10) 2_667 2_666 ?

O007 Nd01 C00H 145.11(11) 2_667 2_666 ?

O008 Nd01 O004 111.09(9) . 2_666 ?

O008 Nd01 O00B 74.83(11) . 2_666 ?

O008 Nd01 C00H 93.61(11) . 2_666 ?

O009 Nd01 O004 119.95(10) . 2_666 ?

O009 Nd01 O005 67.85(11) . . ?

O009 Nd01 O008 128.96(10) . . ?

O009 Nd01 O00B 140.93(11) . 2_666 ?

O009 Nd01 C00H 133.85(11) . 2_666 ?

O00A Nd01 O004 73.99(10) . 1_456 ?

O00A Nd01 O004 67.37(10) . 2_666 ?

O00A Nd01 O005 136.22(11) . . ?

O00A Nd01 O007 71.25(11) . 2_667 ?

O00A Nd01 O008 67.02(10) . . ?

O00A Nd01 O009 133.48(11) . . ?

O00A Nd01 O00B 81.62(11) . 2_666 ?

O00A Nd01 C00H 74.18(11) . 2_666 ?

O00B Nd01 O004 49.87(9) 2_666 2_666 ?

O00B Nd01 C00H 24.61(10) 2_666 2_666 ?

Nd01 O004 Nd01 117.50(11) 1_654 2_666 ?

C00H O004 Nd01 91.7(2) . 2_666 ?

C00H O004 Nd01 150.5(3) . 1_654 ?

Nd01 O005 H00A 109.5 . . ?

Nd01 O005 H00B 109.4 . . ?

H00A O005 H00B 104.4 . . ?

C00G O006 Nd01 160.0(3) . . ?

C00G O007 Nd01 107.6(3) . 2_667 ?

Nd01 O008 H00C 109.4 . . ?

Nd01 O008 H00D 109.4 . . ?

H00C O008 H00D 104.5 . . ?

Nd01 O009 H00E 109.7 . . ?

Nd01 O009 H00F 110.4 . . ?

H00E O009 H00F 118.9 . . ?

Nd01 O00A H00G 109.6 . . ?

Nd01 O00A H00H 109.7 . . ?

H00G O00A H00H 104.3 . . ?

C00H O00B Nd01 95.8(2) . 2_666 ?

C00I N00C C00L 125.3(4) . . ?

C00K N00C C00I 108.9(4) . . ?

C00K N00C C00L 125.8(4) . . ?

H00P O00D H00Q 104.5 . . ?

C00K N00E C00J 125.2(4) . . ?

C00K N00E C00M 108.4(4) . . ?

C00M N00E C00J 126.4(4) . . ?

H00R O00F H00S 109.5 . . ?

O006 C00G C00J 116.2(4) . . ?

O007 C00G O006 124.1(4) . . ?

O007 C00G C00J 119.6(4) . . ?

O004 C00H Nd01 62.9(2) . 2_666 ?

O004 C00H C00L 116.0(3) . . ?

O00B C00H Nd01 59.6(2) . 2_666 ?

O00B C00H O004 122.3(4) . . ?

O00B C00H C00L 121.6(3) . . ?

C00L C00H Nd01 172.4(3) . 2_666 ?

N00C C00I H00I 126.8 . . ?

C00M C00I N00C 106.5(4) . . ?

C00M C00I H00I 126.8 . . ?

N00E C00J C00G 112.1(4) . . ?

N00E C00J H00J 109.2 . . ?

N00E C00J H00K 109.2 . . ?

C00G C00J H00J 109.2 . . ?

C00G C00J H00K 109.2 . . ?

H00J C00J H00K 107.9 . . ?

N00C C00K H00L 125.7 . . ?

N00E C00K N00C 108.6(4) . . ?

N00E C00K H00L 125.7 . . ?

N00C C00L C00H 113.2(3) . . ?

N00C C00L H00M 108.9 . . ?

N00C C00L H00N 108.9 . . ?

C00H C00L H00M 108.9 . . ?

C00H C00L H00N 108.9 . . ?

H00M C00L H00N 107.8 . . ?

N00E C00M H00O 126.2 . . ?

C00I C00M N00E 107.6(4) . . ?

C00I C00M H00O 126.2 . . ?

loop_

_geom_hbond_atom_site_label_D

_geom_hbond_atom_site_label_H

_geom_hbond_atom_site_label_A

_geom_hbond_distance_DH

_geom_hbond_distance_HA

_geom_hbond_distance_DA

_geom_hbond_angle_DHA

_geom_hbond_site_symmetry_A

_geom_hbond_publ_flag

O005 H00A O00F 0.85 1.94 2.689(6) 145.4 . yes

O005 H00B Cl02 0.85 2.29 3.132(3) 168.1 . yes

O008 H00C O00D 0.85 1.88 2.734(5) 175.3 . yes

O009 H00E Cl02 0.86 2.39 3.165(3) 150.4 2_657 yes

O009 H00F O008 0.98 1.79 2.758(4) 172.9 2_667 yes

O00A H00G O00D 0.85 1.94 2.726(5) 153.4 2_577 yes

O00A H00H O005 0.85 1.95 2.799(5) 175.4 2_567 yes

O00D H00P Cl03 0.85 2.24 3.060(4) 162.0 . yes

O00D H00Q Cl02 0.85 2.35 3.165(4) 160.7 1_465 yes

O00F H00S Cl03 0.85 2.27 3.107(5) 169.8 2_666 yes

C00L H00N O007 0.97 2.70 3.180(5) 110.8 2_766 yes

loop_
_geom_torsion_atom_site_label_1
_geom_torsion_atom_site_label_2
_geom_torsion_atom_site_label_3
_geom_torsion_atom_site_label_4
_geom_torsion
_geom_torsion_site_symmetry_1
_geom_torsion_site_symmetry_2
_geom_torsion_site_symmetry_3
_geom_torsion_site_symmetry_4
_geom_torsion_publ_flag
Nd01 O004 C00H Nd01 172.6(5) 1_654 . . 2_666 ?
Nd01 O004 C00H O00B 177.6(3) 1_654 . . . ?
Nd01 O004 C00H O00B 5.0(4) 2_666 . . . ?
Nd01 O004 C00H C00L -171.6(3) 2_666 . . . ?
Nd01 O004 C00H C00L 1.0(7) 1_654 . . . ?
Nd01 O006 C00G O007 179.6(6) . . . . ?
Nd01 O006 C00G C00J 0.7(10) . . . . ?
Nd01 O007 C00G O006 -12.5(5) 2_667 . . . ?
Nd01 O007 C00G C00J 166.4(3) 2_667 . . . ?
Nd01 O00B C00H O004 -5.2(4) 2_666 . . . ?
Nd01 O00B C00H C00L 171.2(3) 2_666 . . . ?
O004 C00H C00L N00C -168.3(3) . . . . ?
O006 C00G C00J N00E 171.4(4) . . . . ?
O007 C00G C00J N00E -7.6(6) . . . . ?
O00B C00H C00L N00C 15.2(6) . . . . ?
N00C C00I C00M N00E 0.2(7) . . . . ?
C00I N00C C00K N00E 0.1(6) . . . . ?
C00I N00C C00L C00H 108.5(5) . . . . ?
C00J N00E C00K N00C -179.8(4) . . . . ?
C00J N00E C00M C00I 179.6(5) . . . . ?
C00K N00C C00I C00M -0.2(6) . . . . ?

C00K N00C C00L C00H -71.8(6) . . . . ?

C00K N00E C00J C00G -116.8(5) . . . . ?

C00K N00E C00M C00I -0.1(7) . . . . ?

C00L N00C C00I C00M 179.5(5) . . . . ?

C00L N00C C00K N00E -179.6(4) . . . . ?

C00M N00E C00J C00G 63.4(7) . . . . ?

C00M N00E C00K N00C 0.0(6) . . . . ?

#END
