# Supplementary material for: Temperature-controlled optical response characteristic of an ionic liquid-based neodymium complex
Source: Front Chem. 2026 Aug 21;14:1888956. doi: 10.3389/fchem.2026.1888956 (PMC13541709; doi:10.3389/fchem.2026.1888956)

Temperature-Controlled Optical Response Characteristic of an Ionic Liquid-Based Neodymium Complex

Shiqi Zhao[a], Boxiao Zhang[a], Wenjie Song[a], Xinnuo Zhao[a], Chunyan Zhou[b*], Yibo Wang[a*]

[a]*Department of Chemistry, Beijing Technology and Business University, Beijing 100048, China*

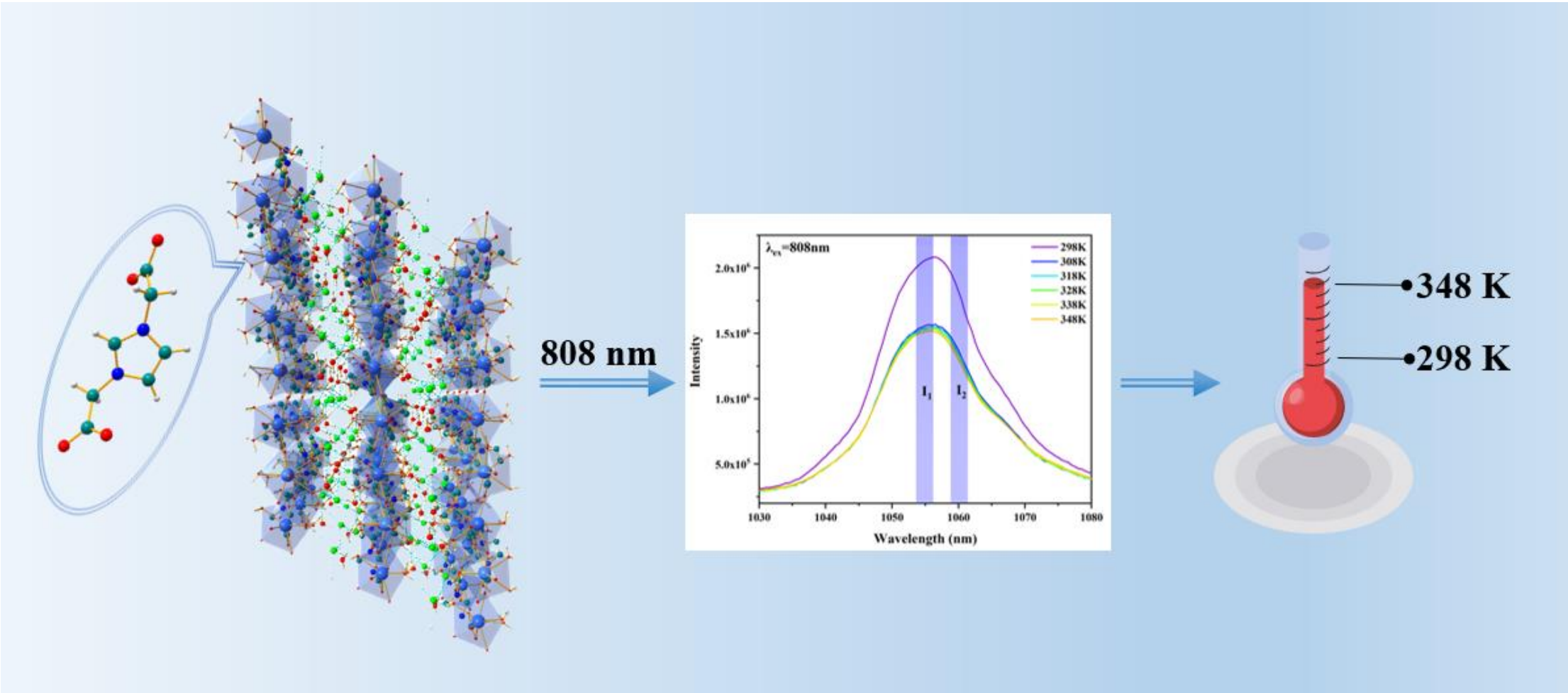

Supplement: Supplementary file 2 [file DataSheet2.pdf]
