# Supplementary material for: Temperature-controlled optical response characteristic of an ionic liquid-based neodymium complex
Source: Front Chem. 2026 Aug 21;14:1888956. doi: 10.3389/fchem.2026.1888956 (PMC13541709; doi:10.3389/fchem.2026.1888956)
Supplement: Supplementary file 3 [file DataSheet3.pdf]

Temperature-Controlled Optical Response Characteristic of an Ionic Liquid-Based Neodymium Complex

Shiqi Zhao[a], Boxiao Zhang[a], Wenjie Song[a], Xinnuo Zhao[a], Chunyan Zhou[b*], Yibo Wang[a*]

[a]*Department of Chemistry, Beijing Technology and Business University, Beijing 100048, China*

[b]*First Affiliated Hospital of Dalian Medical University, Dalian, 116011, China*

Corresponding Authors

[*]*E-mail address: wangyb@th.btbu.edu.cn (Y. Wang)*

Supporting Figures and Tables

Table S1. Reaction conditions and product morphology of $\{[Nd(C_7H_7N_2O_4)(H_2O)_4]Cl_2\cdot 2H_2O\}_n$

Runs	Molar ratio of $NdCl_3\cdot 6H_2O$/IMDC	NaOH (mL)	Volume ratio of $CH_3CH(OH)CH_3/CHCl_3$	Product morphology
1	2:1	0.3	5:5	Purple transparent polycrystalline
2	2:1	0.3	4:6	Flaky bulk polycrystals
3	2:1	0.3	6:4	Fine broken crystals and crystal clustered particles
4	2:1	0.4	5:5	Purple transparent blocky single crystal
5	1:1	0.4	4:6	Purple crystalline powder
6	1:1	0.4	6:4	Purple polycrystals
7	1:1.5	0.5	5:5	Purple broken small polycrystals
8	1:1.5	0.5	4:6	Bulk polycrystals
9	1:1.5	0.5	6:4	Fine purple polycrystals

Table S2. The Crystallographic Information File (CIF) for $\{[Nd(C_7H_7N_2O_4)(H_2O)_4]Cl_2 \cdot 2H_2O\}_n$

Compound	$\{[Nd(C_7H_7N_2O_4)(H_2O)_4]Cl_2 \cdot 2H_2O\}_n$
Empirical formula	$C_7H_{19}O_{10}N_2NdCl_2$
Formula weight	506.38
Temperature/K	295.00 K
Crystal system	Triclinic
Space group	P-1
a/Å	8.9977(5)
b/Å	9.2732(5)
c/Å	11.5128(6)
α/°	89.849(2)
β/°	76.887(2)
γ/°	62.216(2)
Volume/Å^3	821.74(8)
Z	2
ρ_{calc}g/cm^3	2.047
μ/mm^{-1}	19.094
F(000)	498.0
Crystal size/mm^3	0.12×0.1×0.1
Radiation	GaKα(*λ*=1.34139)
2θ range for data collection/°	9.946 to 114.132
Index ranges	-11≤h≤11, -11≤k≤11, -14≤l≤14
Reflections collected	13873
Independent reflections	3348 [R_{int} = 0.0775, R_{sigma} = 0.0609]
Data/restraints/parameters	3348/0/209
Goodness-of-fit on F^2	1.085
Final R indexes [I>=2σ (I)]	R_1 = 0.0365, wR_2 = 0.0864
Final R indexes [all data]	R_1 = 0.0381, wR_2 = 0.0872
Largest diff. peak/hole/ e Å^{-3}	1.32/-1.43

Table S3. Selected bond lengths [Å] for {[Nd($C_7H_7N_2O_4$)(H_2O)$_4$]Cl_2·2H_2O}$_n$

Nd01-O004^1	2.642(3)	Nd01-O008	2.564(3)
Nd01-O004^2	2.493(3)	Nd01-O009	2.495(3)
Nd01-O005	2.554(3)	Nd01-O00A	2.465(3)
Nd01-O006	2.416(3)	Nd01-O00B^1	2.570(3)
Nd01-O007^3	2.481(3)		

Symmetry operation: ^{1}1-X, 1-Y, 1-Z; ^{2}-1+X, +Y, 1+Z; ^{3}1-X, 1-Y, 2-Z

Table S4. Selected bond angles [°] for {[Nd($C_7H_7N_2O_4$)(H_2O)$_4$]Cl_2·2H_2O}$_n$

O004^1-Nd01-O004^2	62.50(11)	O007^3-Nd01-O005	138.88(11)
O004^1-Nd01-O005	82.15(10)	O007^3-Nd01-O008	77.24(11)
O004^1-Nd01-O008	138.91(10)	O007^3-Nd01-O009	71.73(11)
O004^1-Nd01-O009	71.89(10)	O007^3-Nd01-O00B^2	146.87(10)
O004^1-Nd01-O00B^2	112.35(10)	O008-Nd01-O004^2	111.09(9)
O005-Nd01-O004^2	69.12(10)	O008-Nd01-O00B^2	74.83(11)
O005-Nd01-O008	136.13(10)	O009-Nd01-O004^2	119.95(10)
O005-Nd01-O00B^2	74.22(11)	O009-Nd01-O005	67.85(11)
O006-Nd01-O004^2	123.12(10)	O009-Nd01-O008	128.96(10)
O006-Nd01-O004^1	148.32(9)	O00A-Nd01-O004^2	67.37(10)
O006-Nd01-O005	73.16(11)	O00A-Nd01-O005	136.22(11)
O006-Nd01-O007^3	107.51(11)	O00A-Nd01-O007^3	71.25(11)
O006-Nd01-O008	71.47(10)	O00A-Nd01-O008	67.02(10)
O006-Nd01-O009	80.46(10)	O00A-Nd01-O009	133.48(11)
O006-Nd01-O00A	137.67(11)	O00A-Nd01-O00B^2	81.62(11)
O006-Nd01-O00B^2	80.02(10)	O00B^2-Nd01-O004^2	49.87(9)
O007^3-Nd01-O004^2	128.94(9)	Nd01^4-O004-Nd01^2	117.50(11)
O007^3-Nd01-O004^1	78.51(10)	O009-Nd01-O00B2	140.93(11)

Symmetry operation: ^{1}-1+X, +Y, 1+Z; ^{2}1-X, 1-Y, 1-Z; ^{3}1-X, 1-Y, 2-Z; ^{4}1+X, +Y, -1+Z

Table S5. H-bonds for $\{[Nd(C_7H_7N_2O_4)(H_2O)_4]Cl_2{\cdot}2H_2O\}_n$

D	H	A	d(D-H)/Å	d(H-A)/Å	d(D-A)/Å	D-H-A/°
O005	H00A	O00F	0.85	1.94	2.689(6)	145.4
O005	H00B	Cl02	0.85	2.29	3.132(3)	168.1
O008	H00C	O00D	0.85	1.88	2.734(5)	175.3
O009	H00E	Cl02[1]	0.86	2.39	3.165(3)	150.4
O009	H00F	O008[2]	0.98	1.79	2.758(4)	172.9
O00A	H00G	O00D[3]	0.85	1.94	2.726(5)	153.4
O00A	H00H	O005[4]	0.85	1.95	2.799(5)	175.4
O00D	H00P	Cl03	0.85	2.24	3.060(4)	162.0
O00D	H00Q	Cl02[5]	0.85	2.35	3.165(4)	160.7
O00F	H00S	Cl03[6]	0.85	2.27	3.107(5)	169.8

Symmetry operation: [1]1-X, -Y, 2-Z; [2]1-X, 1-Y, 2-Z; [3]-X, 2-Y, 2-Z; [4]-X, 1-Y, 2-Z; [5]-1+X, 1+Y, +Z;

[6]1-X, 1-Y, 1-Z; [7]2-X, 1-Y, 1-Z

Temperature298K

Deconvolution	y_0	x_c	A	w
Fit Peak 1	274409.49708	1054.5766	18549300	14.45402
σ1	2498.00291	0.05451	637198.15758	0.19111
Fit Peak 2	274409.49708	1060.73329	20242300	29.22131
σ2	2498.00291	0.19745	618021.02207	0.48795

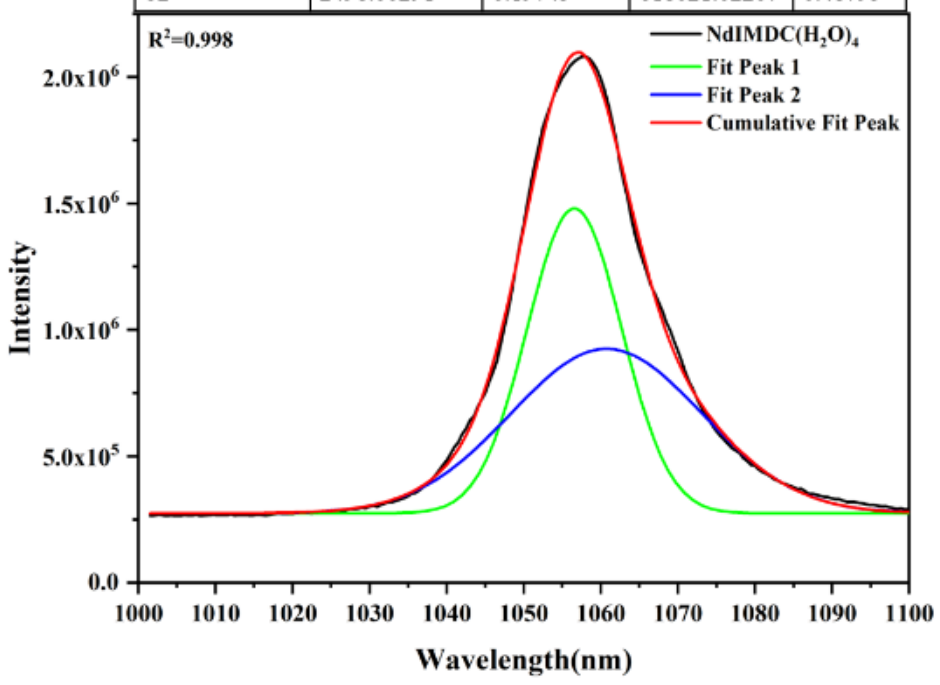

Temperature308K

Deconvolution	y_0	x_c	A	w
Fit Peak 1	271677.16046	1054.80564	12477900	14.07272
σ1	1773.98671	0.05836	447809.81735	0.19741
Fit Peak 2	271677.16046	1060.28784	15346400	28.19914
σ2	1773.98671	0.19311	441574.53192	0.419

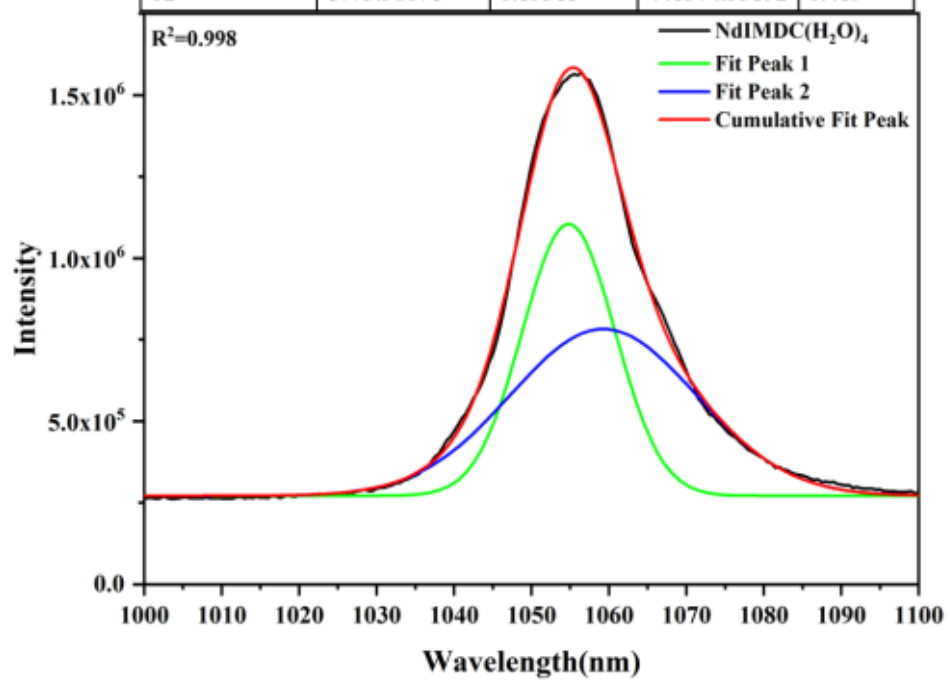

Temperature318K

Deconvolution	y_0	x_c	A	w
Fit Peak 1	270476.79595	1054.7618	12194000	14.00991
σ1	1684.05536	0.05482	411676.97859	0.1868
Fit Peak 2	270476.79595	1060.17953	15421600	28.39838
σ2	1684.05536	0.17739	405084.70748	0.39848

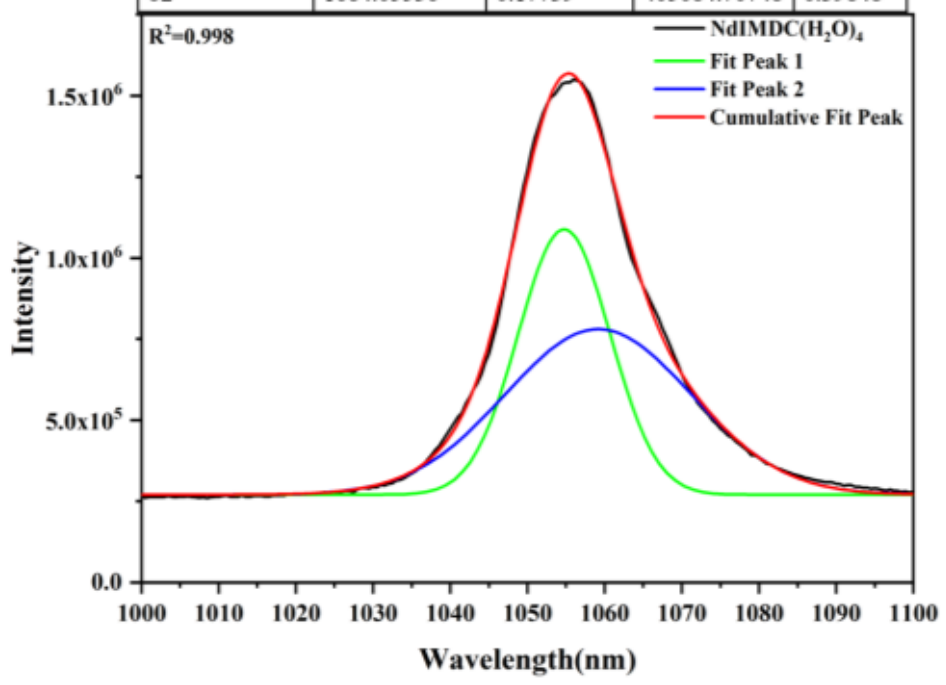

Temperature328K

Deconvolution	y_0	x_c	A	w
Fit Peak 1	269878.01216	1054.72864	11957300	13.91768
σ1	1576.17163	0.05061	367536.62949	0.17206
Fit Peak 2	269878.01216	1060.20221	15512000	28.56634
σ2	1576.17163	0.16227	362762.51126	0.36638

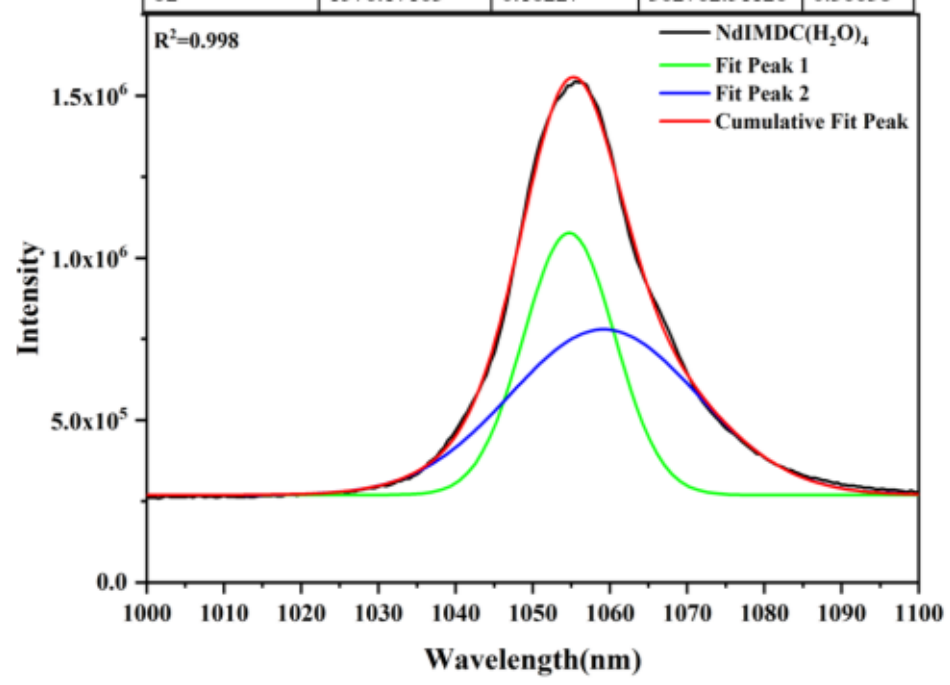

Temperature338K

Deconvolution	y_0	x_c	A	w
Fit Peak 1	272007.88552	1054.59993	11231500	13.6569
σ1	1484.81455	0.04913	334310.93551	0.16627
Fit Peak 2	272007.88552	1060.10838	15937200	28.29725
σ2	1484.81455	0.14648	331482.23761	0.32629

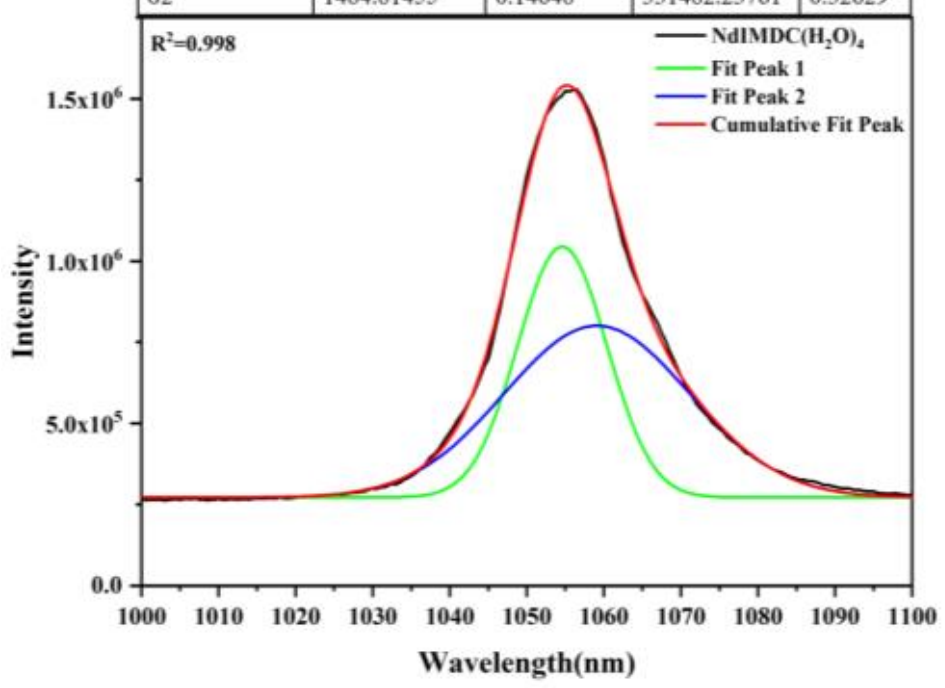

Temperature348K

Deconvolution	y_0	x_c	A	w
Fit Peak 1	274137.03176	1054.55407	11058200	13.59643
σ1	1381.9041	0.04483	292354.41386	0.15066
Fit Peak 2	274137.03176	1060.20182	16081600	28.60732
σ2	1381.9041	0.13344	292021.51395	0.29602

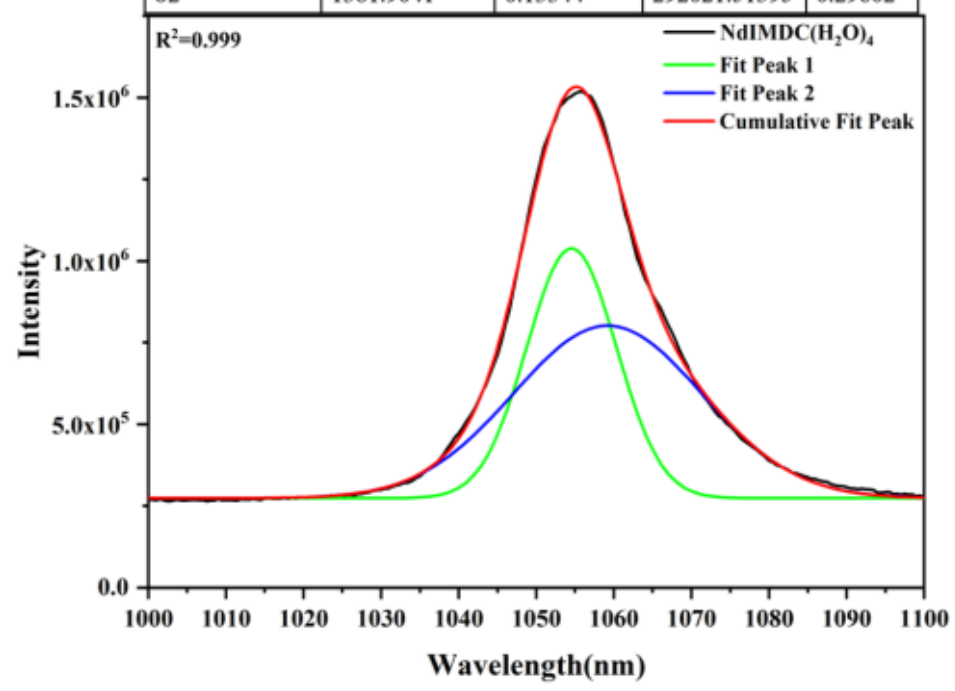

Figure S1. Temperature-dependent emission deconvolution curves for $\{[Nd(C_7H_7N_2O_4)(H_2O)_4]Cl_2\cdot 2H_2O\}_n$ at different temperatures (Fit Peak 1 = 1054 nm； Fit Peak 2 = 1060 nm)

Table S6. Comparison of neodymium-based materials used as luminescent thermometer.

Materials	λ_{ex} (nm)	λ_{em} (nm)	Temperature Range (K)	S_r % K^{-1}	ΔT (K)	Structural Features	Reference
YVO_4:Nd 4.8 at.%	808	1063	298-333	0.25	——	Nanoparticles	[1]
Nd^{3+}:LaF_3 5.6 at.%	808	861-885	293-323	0.4	0.7	Nanoparticles	[2]
LaF_3:Nd	808	870	298-348	0.26	——	Nanoparticles	[3]
YAG:Nd	808	940	288-343	0.15	——	Nanoparticles	[4]
$Nd_{0.890}Yb_{0.110}(BTC)(H_2O)_6$	580	950-1100	12-220	0.8	0.7	CPs	[5]
LaOCl:Nd^{3+}/LaOCl	808	1060	278-333	0.26	——	Nanoparticles	[6]
$La_{0.999}Nd_{0.001}PO_4$	690	750-940	303-348	1.56	——	Nanocrystals	[7]
$Nd_{0.95}Yb_{0.05}$BPTC	808	975-1057	293-323	0.94	0.02	Ln-MOFs	[8]
NdBDC	808	1049-1056	298-333	0.13	——	Ln-MOFs	[9]
NdIMDC	808	1049-1059	298-348	0.618	0.29	Ln-MOCPs	This work

REFERENCE

[1] Kolesnikov, I. E., Golyeva, E. V., Kalinichev, A. A., Kurochkin, M. A., Lähderanta, E., and Mikhailov, M. D. (2017). Nd^{3+} single doped YVO_4 nanoparticles for sub-tissue heating and thermal sensing in the second biological window. *Sensors and Actuators B: Chemical* 243, 338–345. doi: 10.1016/j.snb.2016.12.005

[2] Rocha, U., Jacinto, C., Kumar, K. U., López, F. J., Bravo, D., Solé, J. G., et al. (2016). Real-time deep-tissue thermal sensing with sub-degree resolution by thermally improved Nd^{3+}:LaF_3 multifunctional nanoparticles. *Journal of Luminescence* 175, 149–157. doi: 10.1016/j.jlumin.2016.02.034

[3] Carrasco, E., Del Rosal, B., Sanz-Rodríguez, F., De La Fuente, Á. J., Gonzalez, P. H., Rocha, U., et al. (2015). Intratumoral Thermal Reading During Photo-Thermal Therapy by Multifunctional Fluorescent Nanoparticles. *Adv. Funct. Materials* 25, 615–626. doi: 10.1002/adfm.201403653

[4] Benayas, A., Del Rosal, B., Pérez-Delgado, A., Santacruz-Gómez, K., Jaque, D., Hirata, G. A., et al. (2015). Nd:YAG Near-Infrared Luminescent Nanothermometers. *Advanced Optical Materials* 3, 687–694. doi: 10.1002/adom.201400484

[5] Oggianu, M., Mameli, V., Hernández-Rodríguez, M. A., Monni, N., Souto, M., Brites, C. D. S., et al. (2024). Insights into Nd^{III} to Yb^{III} Energy Transfer and Its Implications in Luminescence Thermometry. *Chem. Mater.* 36, 3452–3463. doi: 10.1021/acs.chemmater.4c00362

[6] Renero-Lecuna, C., Herrero, A., Jimenez De Aberasturi, D., Martínez-Flórez, M., Valiente, R., Mychinko, M., et al. (2021). Nd^{3+} -Doped Lanthanum Oxychloride Nanocrystals as Nanothermometers. *J. Phys. Chem. C* 125, 19887–19896. doi: 10.1021/acs.jpcc.1c05828

[7] Suta, M., Antić, Ž., Đorđević, V., Kuzman, S., Dramićanin, M. D., and Meijerink, A. (2020). Making Nd^{3+} a Sensitive Luminescent Thermometer for Physiological Temperatures—An Account of Pitfalls in Boltzmann Thermometry. *Nanomaterials* 10, 543. doi: 10.3390/nano10030543

[8] Gu, C., Ding, Y., Quan, X., Gong, M., Yu, J., Zhao, D., et al. (2021). Near-infrared luminescent Nd^{3+}/Yb^{3+}-codoped metal–organic framework for ratiometric temperature sensing in physiological range. *Journal of Rare Earths* 39, 1024–1030. doi: 10.1016/j.jre.2020.07.011

[9] Da Silva, K. R. M., Viana, R. S., Dos Santos, M. V., Caiut, J. M. A., Silva, U. R., and Barbosa, C. D. A. E. S. (2025). Nanostructured neodymium-based metal-organic framework for near-infrared luminescent temperature sensor. *Materials Letters* 401, 139206. doi: 10.1016/j.matlet.2025.139206
